# Metabolism‐Based Molecular Subtyping Endows Effective Ketogenic Therapy in p53‐Mutant Colon Cancer

**DOI:** 10.1002/advs.202201992

**Published:** 2022-08-28

**Authors:** Meng Tang, Hui Xu, Hongyan Huang, Hao Kuang, Chenxi Wang, Qinqin Li, Xin Zhang, Yizhong Ge, Mengmeng Song, Xi Zhang, Ziwen Wang, Chaobing Ma, Jinlin Kang, Wanfang Zhang, You Wang, Bo Zhang, Xiaowei Zhang, Yongbing Chen, Minghua Cong, Gerry Melino, Xiaobin Wang, Fuxiang Zhou, Qiang Sun, Hanping Shi

**Affiliations:** ^1^ Department of Radiation and Medical Oncology Hubei Key Laboratory of Tumor Biological Behaviors Hubei Clinical Cancer Study Center Zhongnan Hospital of Wuhan University Wuhan 430071 China; ^2^ Department of Gastrointestinal Surgery/ Department of Clinical Nutrition Beijing Shijitan Hospital Capital Medical University Beijing 10038 China; ^3^ Key Laboratory of Cancer FSMP for State Market Regulation Beijing 100038 China; ^4^ Laboratory of Cell Engineering, Institute of Biotechnology Research Unit of Cell Death Mechanism, 2021RU008 Chinese Academy of Medical Science 20 Dongda Street Beijing 100071 China; ^5^ Comprehensive Oncology Department National Cancer Center/Cancer Hospital Chinese Academy of Medical Sciences and Peking Union Medical College Beijing 100021 China; ^6^ Department of Oncology Beijing Shijitan Hospital Capital Medical University Beijing 10038 China; ^7^ Department of Radiation Oncology Sichuan Cancer Hospital Chengdu 610041 China; ^8^ Department of Pediatric Hematology and Oncology Xinhua Hospital Affiliated to Shanghai Jiaotong University School of Medicine Shanghai 200092 China; ^9^ Department of Experimental Medicine TOR University of Rome“Tor Vergata” Rome 50‐00133 Italy; ^10^ Department of Population Family and Reproductive Health Johns Hopkins University Bloomberg School of Public Health; and Department of Pediatrics Johns Hopkins University School of Medicine Baltimore Maryland 21287 USA

**Keywords:** colon cancer, ketogenic therapy, metabolic subtype, OXCT1, p53

## Abstract

Although targeting cancer metabolism is a promising therapeutic strategy, clinical success depends on accurate molecular and metabolic subtyping. Here, this study reports two metabolism‐based molecular subtypes associated with the ketogenic treatment of colon cancer: glycolytic (glycolysis^+^/ketolysis^−^) and ketolytic (glycolysis^+^/ketolysis^+^), which are manifested by distinct profiles of metabolic enzymes and mitochondrial dysfunction, and by different responses to ketone‐containing interventions in vitro and in vivo. Notably, the glycolytic subtype is able to be transformed into the ketolytic subtype in p53‐mutated tumors upon glucose limitation, rendering resistance to ketogenic therapy associated with upregulation of ketolytic enzymes, such as OXCT1 by mutant p53. The allosteric activator of mutant p53 effectively blocks the rewired molecular expression and the reprogrammed metabolism, leading to the suppression of tumor growth. The findings highlight the utility of metabolic subtyping to guide ketogenic therapy in colon cancer and identify mutant p53 as a synthetic lethality target for ketogenic treatment.

## Graphical abstract

Two metabolism‐based molecular subtypes associated with ketogenic treatment of colon cancer are identified: glycolytic (glycolysis^+^/ketolysis^−^) and ketolytic (glycolysis^+^/ketolysis^+^), which are manifested by distinct profiles of metabolic enzymes and mitochondrial dysfunction, and by different responses to ketone‐containing interventions in vitro and in vivo. The findings highlight the utility of metabolic subtyping to guide the ketogenic therapy in colon cancer and identify mutant p53 as a synthetic lethality target for ketogenic treatment.

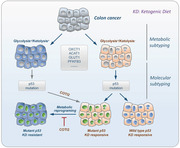

## 1 Introduction

Metabolic reprogramming during tumorigenesis is an essential process in cancer cells. Most solid tumors share the common characteristics of uncontrolled cell proliferation, high demand of energy for cellular growth,^[^ ^1^ ^]^ and increased glucose uptake and dependence on glycolysis.^[^ ^2^ ^]^ The primary example of metabolic reprogramming was discovered by Otto Warburg: tumor cells can shift from oxidative to fermentative metabolism during the process of oncogenesis.^[^ ^3^ ^]^ Recently, the extensive reprogramming of glycolytic activity mediated by posttranslational regulation through phosphorylation has been described;^[^ ^4^ ^]^ for example, tyrosine phosphorylation of enolase (ENO1), pyruvate kinase M (PKM), and phosphoglycerate mutase 1 (PGAM1) and serine phosphorylation of PFKP were significantly increased,^[^ ^5^ ^]^ leading to enzymatic activation and notable changes in the intracellular level of glycolytic metabolites.^[^ ^6^ ^]^ These metabolic features have prompted substantial investigations in both preclinical and clinical trials, in which several metabolism‐targeted agents not only inhibited tumor growth but also improved therapeutic sensitivity.^[^ ^7^ ^]^ One of which is the modification of diet, such as ketogenic diet (KD), a high‐fat, low‐carbohydrate diet with adequate amounts of protein.^[^ ^8^ ^]^ KD mimics the fasting state by decreasing serum glucose levels while simultaneously elevating the levels of ketone bodies as alternative energy.^[^ ^9^ ^]^ It has received great attention because it is safe, low cost, and feasible to implement in both experiments and in clinical settings. Unlike other nutrients, such as essential amino acids, systemic reduction of glucose levels can be achieved by KD.^[^ ^10^ ^]^

However, KD therapy also faces challenges. Previous studies have indicated that the effectiveness of KD as a potential anticancer intervention depends on the functionality of necessary ketolytic enzymes.^[^ ^11^ ^]^ Just as cancer varies greatly in genomic alterations during oncogenesis and proliferation, cancer metabolic reprogramming is also highly heterogeneous under glucose stress,^[^ ^12^ ^]^ part of which is attributed to ketolytic enzyme activities^[^ ^13^ ^]^ or mitochondrial autophagy.^[^ ^14^ ^]^ Therefore, defining metabolic subtypes and elucidating underlying metabolic reprogramming are critical to advance precision KD therapy.

In this study, we hypothesize that in colon cancer, on the one hand, the Warburg effect can be harnessed to serve as an antitumor target for dietary intervention, i.e., creating chronic low glucose stress by a KD; on the other hand, the effectiveness of KD varies by individual cancer's metabolic subtyping, i.e., its ability to adapt to such stress. By screening the baseline molecular profiles in existing datasets of colon cancer patients, we identified different energy metabolic subtypes and investigated their responses to KD treatment. Most remarkably, we found that *p53* ^mt^ cell metabolism could be rewired through nutrient‐sensing pathways to adapt the changes of available energy supplies, leading to resistance to KD treatment. This exploration aimed to target subtype‐specific vulnerabilities in energy metabolism reprogramming and provide combinational strategies to increase the response to ketogenic treatment in cancer.

## 2 Results

### 2.1 Metabolic Heterogeneity in Colon Cancer

**Figure 1:**
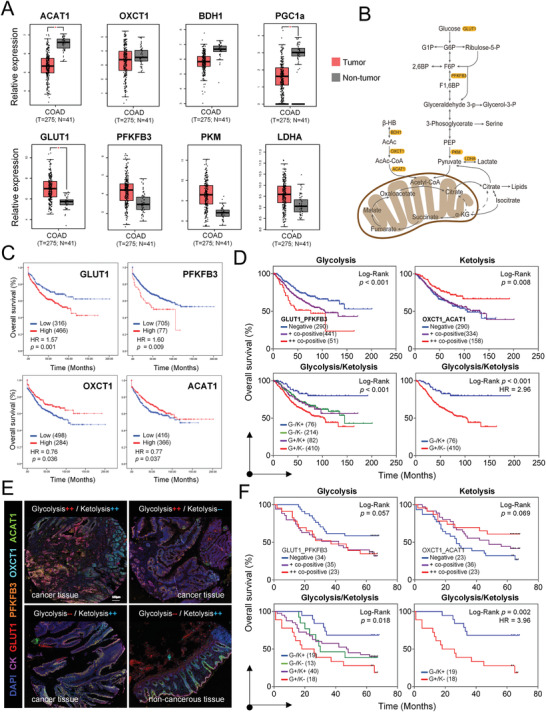
Metabolic heterogeneity and molecular‐based stratification in colon cancer. A) Gene expression scores of metabolic molecules between colon cancer tissues (red) and noncancer tissues (gray) through the GEPIA network (log2FC = 1, which is defined as median (tumor) − median (normal), log scale: log2(TPM+1), *P < 0.01). B) Schematic diagram of glucose and ketone body metabolism. Key molecules are marked with color. C) Kaplan–Meier curve and Cox regression analysis of overall survival (OS) for metabolic molecules of 782 colon cancer patients in the GEO dataset (GSE39582, GSE17536, and GSE17537). D) Kaplan–Meier analysis of overall survival (OS) for glycolytic and ketolytic combined phenotypes at the transcriptional level. (Glycolysis type: GLUT1/PFKFB3, Chi square = 17.33, *P* < 0.001; ketolysis type: OXCT1/ACAT1, Chi square = 7.10, *P* = 0.008; glycolysis/ketolysis: Chi square = 20.37, *P* < 0.001, mOS (G^+^/K^−^) = 102.4 months, mOS (G^−^/K^+^) > 160.8 months). Statistical analyses were generated using the log‐rank test. E) Immunohistochemical staining and multiplexed immunofluorescence (Opal‐7) analysis of glycolytic and ketolytic combined phenotypes in colon carcinoma and adjacent tissues. Blue indicates DAPI (nuclear staining), magenta indicates CK (epithelium), red‐GLUT1, orange‐PFBFK3, cyan‐OXCT1, and green‐ACAT1. F) Kaplan–Meier analysis of overall survival (OS) time for the combined metabolic phenotypes of colon cancer microarray (glycolysis type: GLUT1/PFKFB3, Chi square = 4.18, *P* = 0.057; ketolysis type: OXCT1/ACAT1, Chi square = 5.26, *P* = 0.069; glycolysis/ketolysis: Chi square = 11.30, *P* = 0.018, mOS (G^+^/K^−^) = 27 months, mOS (G^−^/K^+^) > 60 months). Statistical analyses were generated using the log‐rank test.

As the key energy suppliers of cancer cells, the metabolism of glucose and ketone bodies is controlled by a set of key molecules (**Figure** **1**A,B); therefore, we set out to explore the characteristics of colon cancer metabolism by analyzing 16 genes associated with glucose and ketone body metabolic pathways through the Gene Expression Profiling Interactive Analysis (GEPIA) network. As a result, The Cancer Genome Atlas (TCGA) transcriptomics and clinical data were retrieved for 275 patients with colon cancer, along with 41 matched noncancer colon samples. Comparison of the gene expression scores revealed in tumor tissues a decrease of the ketolytic enzyme acetyl‐CoA acetyltransferase (ACAT1) and a trend of decrease in 3‐oxoacid CoA‐transferase 1 (OXCT1) and d‐*β*‐hydroxybutyrate dehydrogenase (BDH1), along with low expression of the oxidative phosphorylation (OXPHOS) factor peroxisome proliferator activated receptor *γ* coactivator‐1*α* (PGC‐1*α*) (Figure 1A, upper panel). The gene expression levels of OXCT1 and ACAT1 in each colon cancer patient varied greatly, suggesting notable differences in ketolytic metabolism. In contrast, most tumor tissues demonstrated higher expression of glucose transporter 1 (GLUT1) (with significance) together with superior levels of its downstream molecules 6‐phosphofructo‐2‐kinase/fructose‐2,6‐bisphosphatase 3 (PFKFB3), pyruvate kinase M (PKM), and lactate dehydrogenase A (LDHA) (trend only) (Figure 1A, lower panel). These results suggested considerable heterogeneity in the metabolism of glucose and ketone bodies in colon cancer.

### 2.2 Molecular Classification of Metabolic Subtypes

To delineate metabolic heterogeneity and identify molecular subtypes of colon cancer that would be sensitive or resistant to ketogenic diet, we selected 782 colon cancer patients from a transcriptome database and randomly divided them into two cohorts: a test cohort consisting of 521 patients with detailed information for clinical and metabolic signature analysis and a validation cohort consisting of 261 patients. Using univariate Cox regression analysis and the least absolute shrinkage and selection operator (LASSO) of the above identified genes, we identified two glycolytic enzymes (GLUT1 and PFKFB3) (Figure S1A, Supporting Information), which, together with the two ketolytic enzymes (OXCT1 and ACAT1), constitute a molecular signature of colon cancer prognosis in the context of ketogenic diet (Figure S1B, Supporting Information). The prognostic value of the four biomarkers, accounting for clinicopathological factors, was assessed by Kaplan‒Meier (KM) curve, univariate Cox regression and multivariate Cox regression analyses. In general, the overexpression of GLUT1 (HR = 1.57, 95% CI: 1.22–2.02, *P* = 0.001) and PFKFB3 (HR = 1.60, 95% CI: 1.13–2.27, *P* = 0.009) indicated poor outcomes, whereas OXCT1 (HR = 0.76, 95% CI: 0.58–0.98, *P* = 0.036) and ACAT1 (HR = 0.77, 95% CI: 0.61–0.99, *P* = 0.037) indicated favorable overall survival for patients with colon cancer (Figure 1C). Next, by considering the combined biomarkers, we defined two molecular metabolism phenotypes of colon cancer: a glycolysis‐proficient phenotype (glycolysis^+^) with either GLUT1 or PFKFB3 overexpression and a ketolysis‐deficient (ketolysis^−^) phenotype by null expression of either OXCT1 or ACAT1. The patients with tumors of either glycolysis^+^ or ketolysis^−^ displayed poor prognosis (Figure 1D, upper panel).

Furthermore, by composite phenotypes, we stratified colon cancer into three prognostic subtypes: the glycolysis^+^/ketolysis^−^ subtype (G^+^/K^−^; GLUT1^high^ or PFKFB3^high^, OXCT1^low^ or ACAT1^low^) demonstrating the worst survival, while the glycolysis^−^/ketolysis^+^ subtype (G^−^/K^+^; GLUT1^low^ and PFKFB3^low^, OXCT1^high^ and ACAT1^high^) demonstrating the most favorable prognosis, and the hybrid subtype (G^−^/K^−^ or G^+^/K^+^) that demonstrates intermediate survival (Figure 1D, lower panel).

### 2.3 Multiplex Staining‐Based Validation of Metabolic Subtyping

To further validate the metabolic molecular signature at the translational level, we employed a multiplex immunohistochemical (mIHC) method, which allows concurrent staining of multiple (up to 7) molecules, to illuminate the expression patterns of the four signature enzymes identified above in a colon cancer tissue microarray (Figure 1E and Figure S1C, Supporting Information). The four molecules are expressed in different cells and frequently do not overlap with each other. Importantly, the same prognostic tendencies were confirmed for the individual metabolic molecules (Figure S1D, Supporting Information), for the two metabolic phenotypes (Figure 1F, upper panel), and for the combined metabolic subtypes (mOS in the G^−^/K^+^ subtype > 60 months vs mOS in the G^+^/K^−^ subtype = 27 months, HR = 3.96, 95% CI: 1.60–9.81, *p* = 0.002) (Figure 1F, lower panel). Taking advantage of the differences in the expression of glycolytic and ketolytic enzymes between colon carcinoma and adjacent tissues, the cancer patients of the G^+^/K^−^ subtype, for enhanced glycolysis and impaired ketolysis, are the ideal population for ketogenic treatment, which accounted for 43.3% (39/90) of the colon cancer patients in the microarray (Figure S1E, Supporting Information) and 52.4% (410/782) of those in the transcriptional dataset.

### 2.4 Metabolic Subtyping Endows Effective Ketogenic Cancer Therapy

**Figure 2:**
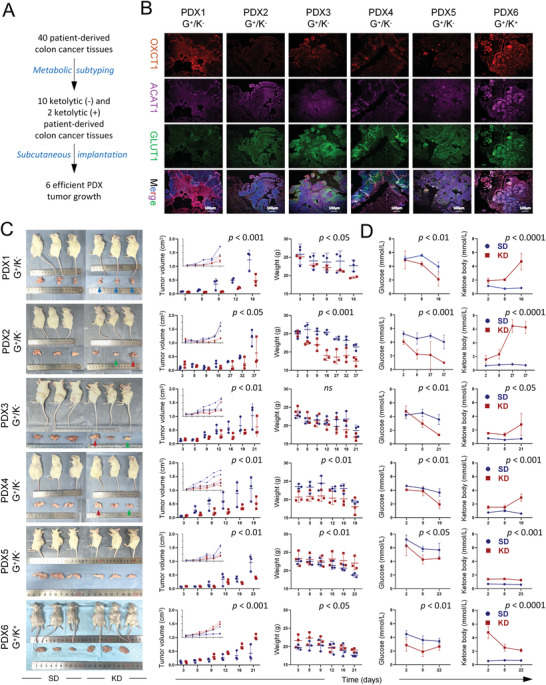
Ketogenic treatment inhibited tumor growth in ketolysis‐deficient PDx models of colon cancer. A) Flowchart of patient‐derived xenograft (PDX) models based on metabolic subtyping. B) Multiplexed immunofluorescence staining (Opal‐4) of colon cancer tissues from corresponding PDX sections. Blue indicates DAPI (nuclear staining), green indicates GLUT1, red‐OXCT1, and magenta‐ACAT1. C) Mouse and tumor images of PDX models after more than two weeks of treatment with the standard diet (SD) and ketogenic diet (KD). Weight and tumor volume were recorded from the third day after grouping treatment with SD or KD until the endpoint. D) Levels of serum glucose and ketone bodies detected from the second day after grouped treatment with SD or KD until the endpoint. Data are presented as the mean ± standard deviation (SD). Two‐way ANOVA was performed between the SD and KD groups (*n* = 3 mice/arm). G^+^/K^−^, glycolysis^+^/ketolysis^−^, G^+^/K^+^, glycolysis^+^/ketolysis^+^.

To examine the effects of metabolic subtyping on ketogenic therapy, we used the patient‐derived xenograft (PDX) tumor model for validation. Totally 40 patient‐derived colon cancer tissues were subjected to metabolic subtyping by multiplex immunostaining of the corresponding PDX sections, ten of which were identified as glycolytic subtypes (G^+^/K^−^) as manifested by low/absent expression of OXCT1 or ACAT1, as well as high expression of GLUT1 or PFKFB3, two of which were identified as ketolytic subtypes (G^+^/K^+^) with all of the four genes overexpressed (**Figure** **2**A,B). Implantation of these 12 lines of patient‐derived colon cancer tissues into immunodeficient mice led to effective tumor growth for six lines, which were subsequently grouped‐fed with a standard diet (SD) and KD when the P3‐generation tumors reached 0.05 cm^3^ in size (Figure 2A,C). Remarkably, compared with the ketolytic subtype (G^+^/K^+^), the growth of all five lines of ketolysis‐deficient PDX tumors was significantly suppressed by a three‐week ketogenic feeding as compared with those in the SD group (Figure 2C), which was correlated with decreased blood glucose (<4.5 mmol L^−1^) along with increased ketone bodies (>1.2 mmol L^−1^) (Figure 2D). Body weight changes between the SD and KD groups were also noticed in the PDX models. Nevertheless, after adjusting the factor of the body weight loss, the tumor‐suppression role of KD intervention was still significant between the two groups (tumor volume/weight at the endpoint).

**Figure 3:**
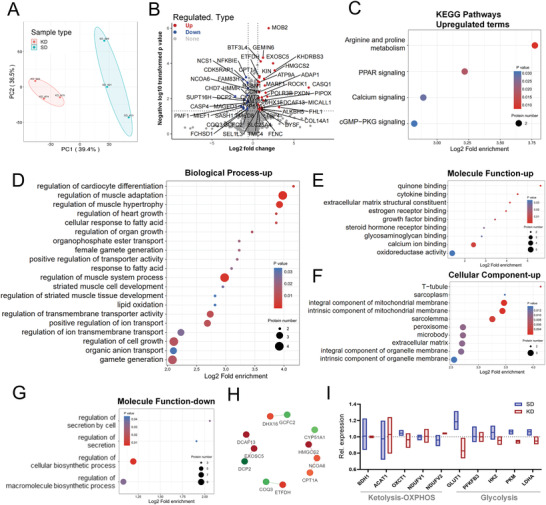
Functional enrichment and cluster analysis of differentially expressed proteins (DEPs) between the KD and SD groups. A) Principal coordinates analysis (PCA) of tumor tissues collected from the KD (left) and SD (right) groups treated for 16 d in PDX1 (*n* = 3 samples/arm). B) Comparison of protein expression in the KD versus SD group (*n* = 3 samples/arm). Differentially expressed proteins are highlighted with red (fold change > 1.5) or blue (fold change < 1/1.5). C) KEGG pathway enrichment of upregulated proteins. D–F) Functional enrichment of upregulated proteins according to GO “biological process,” “molecular function,” and “cellular component” between the KD and SD groups (KD/SD). G) Functional enrichment of downregulated proteins according to GO “molecular function” between the KD and SD groups (KD/SD). H) Protein‒protein interaction (PPI) network of differentially expressed molecules. Red indicates upregulated molecules, and green indicates downregulated molecules. I) Relative protein expression of metabolic molecules associated with ketolytic, OXPHOS, and glycolytic pathways (Rel. expression, the ratio between KD and SD groups, KD/SD).

To explore the molecular impacts of the ketogenic diet, samples fed with either SD or KD were collected from the best responsive PDX tumors and subjected to proteomic analysis. The principal component analysis (PCA) clearly separated the KD group from the SD group, with the KD samples distributed in a more heterogeneous pattern (**Figure** **3**A). Analysis of the differentially expressed molecules identified that the KEGG pathways related to arginine and proline metabolism, PPAR signaling, calcium signaling, and cGMP‐PKG signaling were significantly upregulated upon KD treatment (Figure 3B,C). Gene Ontology (GO) analysis revealed upregulation of molecules involved in the cellular response to fatty acids and lipid oxidation and regulation of transmembrane transporter activity (Figure 3D), consistent with which molecules related to oxidoreductase activity and calcium ion binding (Figure 3E) and those belonging to integral components of the mitochondrial membrane (Figure 3F and Figure S2, Supporting Information) were also upregulated. Conversely, molecules involved in the regulation of cellular and macromolecule biosynthetic processes were downregulated (Figure 3G). Moreover, the deregulated molecules formed several interactive components related to lipid metabolism in mitochondria (Figure 3H). Importantly, under ketogenic treatment, the glycolysis pathway was suppressed, as evidenced by downregulated GLUT1, HK2, PKM, and LDHA (Figure 3I), while the mitochondrial OXPHOS pathway was less affected, which may help ensure ketogenic treatment sensitivity.

### 2.5 Metabolic Characterization of the Glycolytic and Ketolytic Subtypes

**Figure 4:**
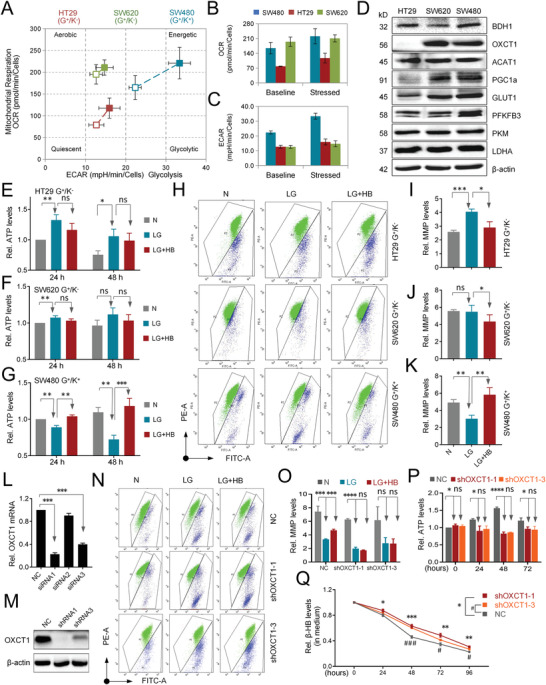
Glycolytic and ketolytic subtypes use glucose and ketone bodies in a different manner. A–C) Measurement of the cell energy phenotype was performed in SW480, HT29, and SW620 cells. Data were normalized by cell numbers. G^+^/K^−^, glycolysis^+^/ketolysis^−^, G^+^/K^+^, glycolysis^+^/ketolysis^+^. D) Western blot of glycolytic and ketogenic molecules in HT29, SW620, and SW480 cells. *β*‐actin was used as a control. E–G) Cellular ATP levels were measured in HT29, SW620, and SW480 cells cultured under normal (N), low glucose (LG), and 5 × 10^−3^ m exogenous *β*‐HB low glucose (LG+HB) conditions for 24 and 48 h. Values were normalized to the cellular protein levels. H–K) Mitochondrial membrane potential (MMP) was detected in HT29, SW620, and SW480 (K) cells cultured under N, LG, and LG+HB conditions, respectively, for 48 h. L) Relative mRNA levels of OXCT1 in 293T siOXCT1 cells (three sequences) and NC cells (control vector of siOXCT1). M) Western blot analysis of OXCT1 in SW480 shOXCT1 cells (shRNA sequence 1 and sequence 3) and NC cells (shOXCT1 control vectors). *β*‐actin was used as an internal control. N,O) MMP was detected in SW480 cells treated with NC (upper panel), shOXCT1‐1 (sequence 1 of shOXCT1), or shOXCT1‐3 (sequence 3 of shOXCT1) (lower panel) and cultured under N, LG, and LG+HB conditions for 48 h. P) Cellular ATP levels were measured in SW480 cells treated with NC, shOXCT1‐1, or shOXCT1‐3 and cultured under conditions containing 5 × 10^−3^ m exogenous *β*‐HB low glucose (LG+HB) for 24, 48, and 72 h. Values were normalized to cellular protein. Q) Relative *β*‐HB levels were measured in SW480 cells treated with NC, shOXCT1‐1, or shOXCT1‐3 and cultured under 5 × 10^−3^ m exogenous *β*‐HB low glucose (LG+HB) conditions for 24, 48, 72, and 96 h. *P < 0.05 compared between the shOXCT1‐1 and NC groups, ^#^ *P* < 0.05 compared between the shOXCT1‐3 and NC groups. Data are presented as the mean ± SD. All of the error bars denote the SD of triplicates. ^#^, *P < 0.05, ^##^, **P < 0.01, ^###^, ***P < 0.001, ****P < 0.0001.

To further investigate the metabolic properties of colon carcinoma, five colon cancer cell lines available in the lab were examined for the identification of the metabolic subtype. Considering the suitability of metabolic subtypes and easy‐to‐culture characteristics, three colon cancer cell lines (SW480, HT29, and SW620), commonly used by other investigators,^[^ ^15^ ^]^ were selected for further study. As the energy phenotype assay showed (**Figure** **4**A), different cells meet discrepant energy demands via mitochondrial respiration or glycolysis. Among them, SW480 cells were identified as energetic cells that are susceptible to changes in the oxygen consumption rate (OCR), an indication of aerobic respiration, and changes in the extracellular acidification rate (ECAR), a readout of the lactic acid produced from aerobic glycolysis (Figure 4B,C). Whereas HT29 cells displayed a relative increase in mitochondria‐driven oxygen consumption and ATP production under mitochondrial stress, SW620 cells exhibited complete mitochondrial stagnancy. Moreover, with a view to the expression of glycolytic and ketolytic enzymes (Figure 4D), the SW480 cell line could be defined as the hybrid subtype of glycolysis^+^/ketolysis^+^, which displayed extremely active glycolysis and oxidative phosphorylation with overexpression of most key enzymes and possessed a ketolytic ability without any deficiencies of BDH1, OXCT1 or ACAT1. HT29 and SW620 cells were classified as the glycolytic subtype of glycolysis^+^/ketolysis^−^ with a deficiency of either OXCT1 or BDH1.

In line with the glycolysis subtype (G^+^/K^−^), supplementing HT29 and SW620 cells with exogenous beta‐hydroxybutyrate (*β*‐HB), the most abundant form of ketone bodies, under glucose‐limiting conditions could not restore the cellular ATP levels or mitochondrial membrane potential (MMP), which was in contrast to the responses of the hybrid subtype (G^+^/K^+^) SW480 cells (Figure 4E–K). To further confirm an essential role of enzyme expression in molecular subtyping and cellular response to metabolites, OXCT1, whose expression was absent in HT29 cells of the glycolysis subtype (G^+^/K^−^), was knocked down in SW480 cells (G^+^/K^+^). As expected, OXCT1 knockdown strongly decreased the ability of cells to utilize ketone bodies, as assayed by the decline in MMP, reduction in intracellular ATP and higher levels of spare ketone bodies in culture (Figure 4L–Q).

### 2.6 Metabolic Reprogramming of Ketolysis: Cells Undergoing Low Glucose Stress

**Figure 5:**
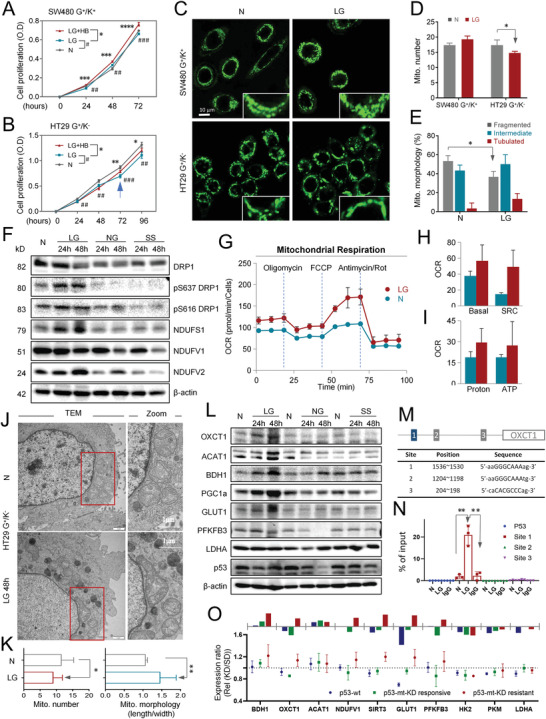
Metabolic reprogramming under glucose‐limiting conditions. A,B) Proliferation curves of SW480 and HT29 cells cultured under N, LG, and LG+HB conditions. Cell numbers were detected by the CCK8 test. Data are presented as the mean ± SD. All of the error bars denote the SD of four replications. *P < 0.05 compared between the LG+HB and LG groups, ^#^ *P* < 0.05 compared between the LG and N groups. C) Mitochondrial morphology of SW480 and HT29 cells cultured under normal (N) and low glucose (LG) conditions for 48 h and stained with MitoTracker (green). D,E) Quantification of mitochondrial numbers and morphology of SW480 and HT29 cells cultured under normal and LG conditions for 48 h. Data are presented as the mean ± SD (30 cells per group). *P < 0.05 compared with the N group. F) Western blot analysis of mitochondrial dynamin‐related proteins and subunits of respiratory chain complex I in HT29 cells cultured under N, LG, nonglucose (NG), and serum starvation (SS) conditions for 24 and 48 h. *β*‐actin was used as an internal control. G–I) Measurement of the OCR in HT29 cells cultured under N and LG conditions for 48 h. Data were normalized by cell numbers. SRC, spare respiratory capacity. J) Representative transmission electron microscopy images of mitochondria in HT29 cells cultured under N and LG conditions for 48 h. K) Quantification of mitochondrial numbers of each cell and morphology (length/width) of each mitochondrion of HT29 cells cultured under N and LG conditions for 48 h. Data are presented as the mean ± SD. Error bars denote the SD of 15 replications (15 cells per group or 15 mitochondria per group). L) Western blot analysis of glycolytic and ketolytic enzymes in HT29 cells cultured under normal, low glucose (LG), nonglucose (NG), and serum starvation (SS) conditions for 24 and 48 h. *β*‐actin was used as an internal control. M) Schematic of the p53 binding motif and sequences in the OXCT1 promoter. N) ChIP‒qPCR showing enrichment of p53 binding to the OXCT1 promoter in HT29 cells cultured under LG conditions for 48 h compared with the control. Signals were normalized with cell numbers. The fold changes were calculated by normalization to the input. O) Relative protein expression of metabolic molecules associated with ketolytic, OXPHOS, and glycolytic pathways in colon cancer tissue derived from PDx models. Blue indicates tissues with wild‐type p53, green indicates KD‐responsive tissues with mutant p53, and red indicates KD‐resistant tissues with mutant p53 (Rel. expression, which is defined as the value of the ratio between the KD and SD groups, KD/SD). Data are presented as the mean ± SD. All of the error bars denote the SD of triplicates. ^#^, *P < 0.05, ^##^, **P < 0.01, ^###^, ***P < 0.001, ****P < 0.0001.

Next, the G^+^/K^+^ SW480 cells and G^+^/K^−^ HT29 cells were examined for their growth under conditions reminiscent of ketogenic therapy, i.e., ®‐HB plus low glucose (LG). As shown in **Figure** **5**A,B, the growth of the two glycolysis^+^ cell lines was significantly compromised upon glucose deprivation (LG) over 72 h. Moreover, under LG conditions, *β*‐HB could restore the growth of ketolysis‐proficient SW480 cells but not ketolysis‐deficient HT29 cells within 48 h, which is in line with expectations. Interestingly, it was noted that the growth inhibition of HT29 cells was partially rescued after 72 h by supplementing *β*‐HB (arrow in Figure 5B), suggesting that metabolic reprogramming took place upon LG stress in HT29 cells, which endowed the ability to utilize *β*‐HB as an energy source to facilitate cell growth. In agreement with this idea, confocal microscopy showed that the number of mitochondria in HT29 cells significantly decreased after 48 h‐LG culture (Figure 5C,D), which took place with reduced fragmentation and increased mitochondrial tubulation (Figure 5C–E), suggesting the occurrence of mitochondrial fusion upon LG stress. Consistently, the expression of dynamin‐related protein 1 (Drp1) and its phosphorylation at Ser‐616 (pS616‐Drp1) were downregulated, whereas Drp1 with phosphorylation at Ser‐637 (pS637‐Drp1), which suppresses mitochondrial fission, was upregulated under glucose‐limiting conditions (Figure 5F). In addition, the subunits of mitochondrial respiratory chain complex I, such as NDUFS1 and NDUFV2, were apparently induced. To further confirm the metabolic adaptation under glucose‐limiting conditions, HT29 cells were subjected to OCR and transmission electron microscopy (TEM) measurements, which indicated proficient mitochondrial oxidative phosphorylation and enhanced mitochondrial fusion (Figure 5G–K).

### 2.7 Metabolic Reprogramming by Mutant p53 Mediated the Upregulation of Ketolytic Molecules

Moreover, LG stress, but not nonglucose (NG) or serum starvation (SS), strongly induced the expression of the major molecules in the ketolysis pathway, including OXCT1, ACAT1, BDH1, and PGC1a as well (Figure 5L), which underlies metabolic reprogramming in HT29 cells (Figure 5B). Since p53 is a key regulator of metabolism that functions primarily at the transcriptional level,^[^ ^16^ ^]^ we, therefore, set to examine its involvement in this context. Due to its rather low expression in HT29 cells (Figure 4D) and requirement for ketolytic activity in SW480 cells (Figure 4L–Q), OXCT1 was selected for validation as a proof of principle. Remarkably, bioinformatic analysis identified three potential binding sites for p53 in the 5′ upstream regulatory region of the OXCT1 coding gene (Figure 5M), of which the first site was confirmed to be bound by p53 that is mutated in HT29 cells (Figure S3A–D, Supporting Information) upon LG stress by chromatin immunoprecipitation (ChIP)‐qPCR assay (Figure 5N). In agreement with the result, the large PDX tumors with mutated p53 in the KD group (p53‐mt‐KD resistant, red arrows in Figure 2C) tended to upregulate the expression of molecules of the ketolysis pathway upon ketogenic treatment, as compare with the paired small PDX tumors (p53‐mt‐KD responsive, green arrows in Figure 2C) or the PDX tumors of wild‐type p53 (p53‐wt, blue arrows in Figure 2C) as analyzed by proteomic profiling (Figure 5O and Figure S3E, Supporting Information, for genotyping) and confirmed by immunohistochemical staining and Western Blot (Figure S4, Supporting Information). Additionally, analysis of the GEPIA database revealed a significant positive correlation between p53 and OXCT1 specifically in the tumor tissues of colon cancer, but not in paracancerous tissues or normal colon tissues (Figure S3F–I, Supporting Information). Together, these data fit with a model that mutant p53 mediating metabolic reprogramming by upregulating ketolytic molecules could confer resistance to ketogenic therapy. For those KD‐sensitive cases with mutant p53, the underlying mechanisms might be associated with the in vivo balance between energy and nutrition, which warrants further investigation.

### 2.8 P53 Activator Reverses Mitochondrial OXPHOS Adaptation

**Figure 6:**
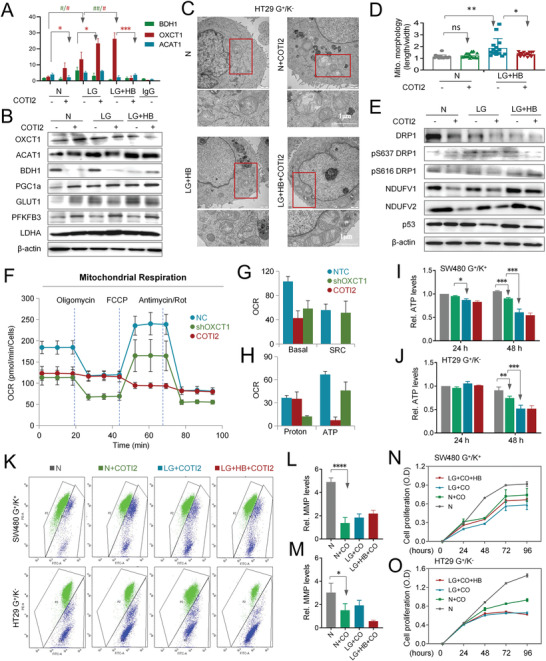
p53 activator reversed metabolic reprogramming under glucose‐limiting conditions. A) ChIP‒qPCR test of p53 enrichment on the BDH1, OXCT1, or ACAT1 promoter in HT29 cells cultured under normal (N), low glucose (LG), and containing 5 × 10^−3^ m exogenous *β*‐HB low glucose (LG+HB) conditions with 5 × 10^−6^ m COTI2 for 24 h or without 5 × 10^−6^ m COTI2 for 48 h. The fold changes were calculated by normalization to the input. Data are presented as the mean ± SD. Error bars denote the SD of triplicates. *P < 0.05 compared between the COTI2 group and non‐COTI2 group, ^#^ *P* < 0.05 compared between the LG, LG+HB group and N group. The green symbol indicates the significance of BDH1, and the red symbol indicates the significance of OXCT1. B) Western blot analysis of glycolytic and ketolytic enzymes in HT29 cells cultured under N, LG, and LG+HB with or without 5 × 10^−6^ m COTI2 conditions for 48 h. *β*‐actin was used as an internal control. C) Representative transmission electron microscopy images of mitochondria in HT29 cells cultured under N and LG+HB conditions with 5 × 10^−6^ m COTI2 conditions for 24 h or without 5 × 10^−6^ m COTI2 conditions for 48 h. D) Quantification of mitochondrial morphology (length/width) of HT29 cells cultured under N and LG+HB conditions with 5 × 10^−6^ m COTI2 conditions for 24 h or without 5 × 10^−6^ m COTI2 conditions for 48 h. Data are presented as the mean ± SD (15 mitochondria per group). E) Western blot analysis of mitochondrial dynamin‐related proteins and subunits of respiratory chain complex I in HT29 cells cultured under N, LG, and LG+HB with or without 5 × 10^−6^ m COTI2 conditions for 48 h. *β*‐actin was used as an internal control. F–H) Measurement of mitochondrial respiration was performed in SW480 cells treated with control vector (NC), shOXCT1, and 5 × 10^−6^ m COTI2 for 24 h. Data were normalized by cell numbers and presented as the mean ± SD. Error bars denote the SD of triplicates. SRC, spare respiratory capacity. I,J) Cellular ATP levels were measured in SW480 and HT29 cells cultured under N, N+COTI2, LG+COTI2, and LG+HB+COTI2 conditions for 24 and 48 h. Values were normalized to the cellular protein levels. Data are presented as the mean ± SD. Error bars denote the SD of triplicates. *P < 0.05 compared within each group. K–M) Mitochondrial membrane potential was detected in SW480 and HT29 cells cultured under normal (N), N+COTI2, LG+COTI2, and LG+HB+COTI2 conditions for 24 h. Data are presented as the mean ± SD. Error bars denote the SD of triplicates. *P < 0.05 compared within each group. N,O) Proliferation curves of SW480 and HT29 cells cultured under N, N+COTI2, LG+COTI2, and LG+HB+COTI2 conditions. Cell numbers were detected by the CCK8 test. Data are presented as the mean ± SD. Error bars denote the SD of four replications. *P < 0.05 compared between the LG+COTI2 and LG+HB+COTI2 groups. ^#^, *P < 0.05, ^##^, **P < 0.01, ^###^, ***P < 0.001, ****P < 0.0001.

Next, we examined the effects of interfering with mutant p53 function by COTI2, an allosteric activator, on metabolic subtyping and ketone body metabolism. As shown in **Figure** **6**A, treatment of p53‐mutant HT29 cells with 5 × 10^−6^ m COTI2 notably reversed the enhanced p53 binding to the site 1 of OXCT1 promoter under ketogenic conditions (significant difference between the LG+HB and LG+HB+CO groups), while there was no significant difference between mutant p53 and BDH1 or ACAT1, respectively. Consistently, exogenous COTI2 inhibited the LG‐induced overexpression of ketolytic molecules over a period of 48 hours, including OXCT1, ACAT1, BDH1, and PGC1a, particularly KD‐activated OXCT1, in both HT29 cells and SW480 cells (Figure 6B and Figure S5A–C, Supporting Information). This was true for cells cultured in media with either a normal dose of glucose (N) or low glucose (LG), which was marginally affected by further addition of exogenous *β*‐HB. These data suggested that both the G^+^/K^−^ HT29 and G^+^/K^+^ SW480 cells were constitutively metabolism‐reprogrammed, and targeted allosterism of mutant p53 could stabilize the subtype as G^+^/K^−^ type, leading to a compromised utility of ketone bodies and a restored responsiveness to ketogenic therapy. Concomitantly, COTI2 treatment also regulated the morphology of mitochondria in a way toward fission (Figure 6C,D and Figure S5D, Supporting Information), as supported by the strikingly downregulated expression of Ps637 DRP1, NDUFV2, and the like, both in HT29 cells and SW480 cells (Figure 6E and Figure S5E,F, Supporting Information). Consistent with this idea, COTI2 treatment suppressed mitochondrial oxidative phosphorylation in a way even stronger than did OXCT1 knockdown (shOXCT1) (Figure 6F–H), which occurred along with reduced intracellular ATP, declined MMP (Figure 6I–M), and impaired production of acetyl‐CoA (Figure S5G,H, Supporting Information) in both SW480 and HT29 cells, and the effects could not be rescued by supplementation with exogenous *β*‐HB. Importantly, COTI2 treatment profoundly suppressed the growth of both SW480 and HT29 cells in either N or LG media, which was not significantly impacted by exogenous *β*‐HB (Figure 6N,O). Thus, functional inhibition of mutant p53 by COTI2 may serve as a feasible way to rewire metabolism subtypes and restore responsiveness to ketogenic therapy.

### 2.9 Targeting Metabolic Reprogramming to Improve the Effectiveness of Ketogenic Treatment

**Figure 7:**
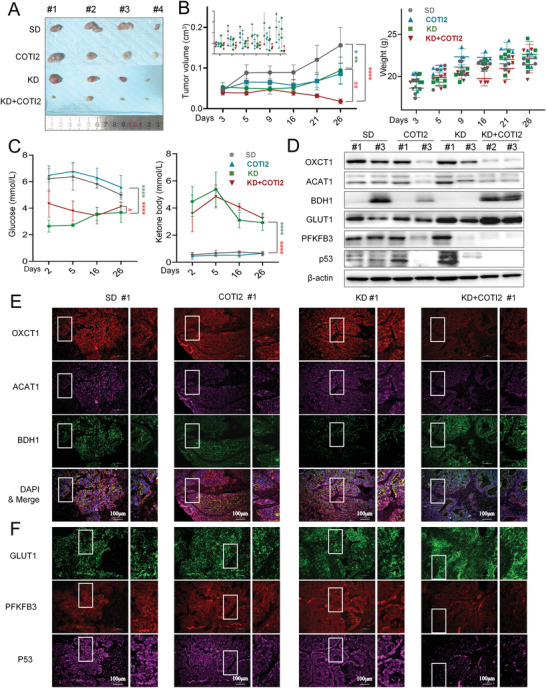
Reversing metabolic reprogramming promotes sensitivity to ketogenic treatment. A,B) Tumor images and volumes of P4‐generation PDX models after a three‐week treatment with the standard diet (SD), ketogenic diet (KD), 10 mg kg^−1^ COTI2 (COTI2), or KD combined with 10 mg kg^−1^ COTI2 (KD+COTI2). C) Levels of serum glucose and ketone bodies detected from the second day after grouped treatment until the endpoint. D) Western blot of glycolytic and ketolytic molecules in the corresponding PDX tumor tissues in the P4 generation. *β*‐actin was used as an internal control. E) Multiplexed immunofluorescence staining (Opal‐4) of colon cancer tissues from corresponding PDX sections. Red indicates OXCT1, magenta‐ACAT1, green‐BDH1, and blue indicates DAPI (nuclear staining). F) Multiplexed immunofluorescence staining (Opal‐4) of colon cancer tissues from corresponding PDX sections. Green indicates GLUT1, red‐PKFKB3, and magenta‐P53. Data are the mean ± SEM, and two‐way ANOVA was performed among each group (*n* = 4 mice/arm). *P < 0.05 compared between the COTI2, KD, or KD+COTI2 group and the SD group, blue asterisk indicates the COTI2 and SD groups, green asterisk indicates the KD and SD groups, red asterisk indicates the KD+COTI2 and SD groups, ^#^ *P* < 0.05 compared between the KD+COTI2 and KD groups. ^#^, *P < 0.05, ^##^, **P < 0.01, ^###^, ***P < 0.001, ****P < 0.0001.

To examine whether targeting mutant p53 could improve the antitumor effect of ketogenic treatment, a cohort of PDX (P4‐generation of PDX3 in Figure 2C, G^+^/K^−^/p53^mt^) and HT29 (G^+^/K^−^/*p53* ^mt^) colon cancer xenografts were fed a KD together with COTI2 agent, respectively. While KD alone displayed tumor‐suppressive effects over a two‐ or three‐week period compared with SD treatment, a combination of KD and COTI2 further inhibited tumor growth (**Figure** **7**A–C and Figure S6A,B, Supporting Information). This is correlated with a profound decrease in proliferation, as evidenced by significant reductions in the expression of Ki67 and PCNA (Figure S6C, Supporting Information), which was conceivably attributed to the blocked metabolic reprogramming by mutant p53 inactivation, as COTI2 feeding completely suppressed the expression of ketolytic OXCT1 induced by KD (Figure 7D–F and Figure S6D, Supporting Information), as identified above (Figure 5O). Together, these data consistently identified mutant p53 as a synthetic lethality target for ketogenic treatment of colon cancer.

## 3 Discussion

**Figure 8:**
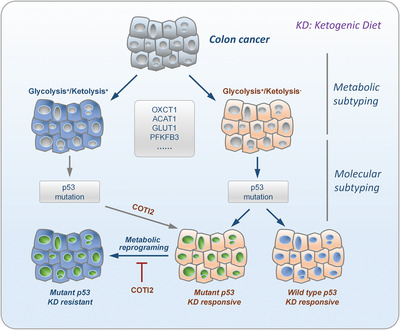
Graphical abstract: Glycolysis^+^/Ketolysis^+^, G^+^/K^+^ (GLUT1^high^ or PFKFB3^high^, OXCT1^high^, and ACAT1^high^); Glycolysis^+^/ketolysis^−^, G^+^/K^−^ (GLUT1^high^ or PFKFB3^high^, OXCT1^low^ or ACAT1^low^); COTI2, an allosteric activator that converts mutant p53 to the wild‐type conformation.

Whereas aberrant metabolism is well known to be pivotal for tumor development and progression, targeting metabolism for cancer therapy turned out to be challenging largely due to high metabolic heterogeneity, which is particularly true for ketogenic therapy. Aiming at this challenge, in this study, we proposed a guideline of metabolism‐based molecular subtyping for effective ketogenic therapy of colon cancer (**Figure** **8**). According to this guideline, colon cancers can be divided into either ketolytic (G^+^/K^+^) or glycolytic (G^+^/K^−^) subtypes based on the expression of OXCT1, ACAT1, GLUT1, and PFKPF3. Meanwhile, tumors were genotyped on the status of p53 by exon sequencing. On one side, glycolytic tumors with wild‐type p53 are supposed to be responsive to KD intervention, whereas those with mutant p53 are recommended to be given KD in combination with allosteric activators of mutant p53, such as COTI2. This helps prevent therapy resistance caused by LG‐induced metabolic reprogramming via mutant p53 that triggers the expression of multiple metabolic genes,^[^ ^17^ ^]^ including OXCT1 reported in this study. On the other side, ketolytic tumors are supposed to be resistant to KD intervention; however, those with mutant p53 might be transformed into KD‐responsive tumors by allosteric activators of mutant p53, such as COTI2. Notably, the feasibility of this guideline warrants careful validation in clinical trials in the near future.

In this study, in vitro mitochondrial reprogramming was investigated under glucose‐limiting and ketogenic culture conditions, but as a patient‐specific multifactorial therapy, the promising antitumor function of the ketogenic diet strictly relies on the created unfavorable metabolic environment for cancer cells. Therefore, the efficiency of in vivo ketogenesis and ketone body utilization regulated by hormones, such as insulin and glucagon, as well as the metabolites of tumors, should be confirmed under KD intervention. In addition, owing to the composition of the ketogenic diet, especially the high fat content, a general concern is that KD may cause deterioration of certain metabolites, such as cholesterol and triglycerides, while some studies point toward an improvement of body composition.^[^ ^18^ ^]^ In this study, body weight changes were observed in mice bearing colon cancer tissues from different patients, and the underlying mechanisms of KD‐induced weight loss, which are associated with the energy–nutrition imbalance in tumors, need further investigation.

Previous studies have reported the bioactive effects of ketone bodies that function as signaling molecules and regulate histone modifications through lysine *β*‐hydroxybutyrylation (Kbhb) by *β*‐hydroxybutyrate (*β*‐HB).^[^ ^19^ ^]^ In addition, histone Kbhb was proven to be enriched in active gene promoters that further upregulated starvation‐responsive metabolic pathways.^[^ ^20^ ^]^ In this study, based on the metabolic phenotypes, the accumulated *β*‐HB showed a function that promotes unrecoverable impairment in mitochondrial membrane potential and cell proliferation, while ketolysis‐deficient cancer cells lost the capacity to adapt to using ketone bodies as highly efficient metabolites. However, the underlying mechanisms of the accumulated *β*‐HB leading to tumor suppression through Kbhb still need further investigation. Furthermore, although four signature molecules (OXCT1, ACAT1, GLUT1, and PFKPF3) were identified in this study to stratify the metabolic subgroups in colon cancer, they may not be the optimal set for other types of cancers. Actually, it is known that cancers initiate multiple metabolic compensations as a result of complicated interactions between cancer cells and microenvironments. In line with this idea, the mutational landscape and the gene expression patterns of glycolytic and ketolytic molecules vary across different cancer types and even different intratumor clones.^[^ ^21^ ^]^ Compelling evidence demonstrates that multiple subtypes define distinct biological and therapeutic vulnerabilities of cancers, providing a de novo clinically applicable molecular taxonomy,^[^ ^22^ ^]^ which warrants a comprehensive understanding of tumor therapeutic resistance to develop better strategies.^[^ ^23^ ^]^ Therefore, a cancer type‐oriented discovery of different metabolic signature sets is preferred for more precise metabolic subtyping.

It is noted that the identification of mutant p53 as a driver of metabolic reprogramming in this study does not deny the potential involvement of other oncogenic genes. In fact, many other oncogenic mutations and/or activated signaling pathways, such as Kras mutation and c‐Myc, are also important regulators of cancer metabolism; thus, exploring their implications in ketogenic therapy may further shed light on novel therapeutic strategies and guidelines.^[^ ^18^ ^]^ For example, constitutive activation of Kras signaling by point mutations in either G12V or G13D was shown to drive metabolic reprogramming to promote the development and progression of multiple cancers, such as lung carcinoma.^[^ ^24^ ^]^ This worked out primarily by increasing the glycolytic activity of cancer cells that consume excessive amounts of glucose to sustain cancer growth. It is conceivable that cells with higher glycolytic activity, such as those with Kras mutations, are more vulnerable to glucose restriction. Thus, a composite molecular subtyping targeting both glycolysis and ketolysis would help define a group of cancer patients who are highly responsive to ketogenic therapy, for whom a modified recipe of KD with a medium level of carbohydrates, which are more tolerable to patients, may also be functional in suppressing tumor growth. In light of this point, this study actually sets a basis for more accurate molecular subtyping toward precision ketogenic therapy of multiple cancer types.

## 4 Experimental Section

### Cell Line and Culture Conditions

The human colon cancer cell lines SW480, HT29, and SW620 were purchased from Procell (Life Science & Technology Co., Ltd.) and normally cultured in DMEM (4.5 g L^−1^ glucose, 4.0 × 10^−3^ m l‐glutamine, without pyruvate) supplemented with 10% fetal bovine serum and 1% penicillin–streptomycin, conventionally placed in a 5% CO_2_ incubator at 37 °C. These cell lines were assessed for mycoplasma contamination, and the test results were negative. For subsequent experiments, the medium was discarded gently, washed twice with PBS, and then treated under specific conditions in a 5% CO_2_ incubator at 37 °C for 24 or 48 h. To establish a cell model of nutrient deficiency or imitate ketogenic conditions, different procedures used in this study were designed as follows:

- Low glucose (LG): low glucose medium (1 g L^−1^ glucose, 4.0 × 10^−3^ m l‐glutamine, without pyruvate) containing 10% fetal serum and 1% penicillin‒streptomycin.
- Nonglucose (NG): glucose‐free medium (0 g L^−1^ glucose, 4.0 × 10^−3^ m l‐glutamine, without pyruvate) containing 10% fetal serum and 1% penicillin‒streptomycin.
- Serum starvation (SS): serum‐free DMEM (4.5 g L^−1^ glucose, 4.0 × 10^−3^ m l‐glutamine, without pyruvate) containing 1% penicillin‒streptomycin.
- Ketogenic condition (LG+HB): low glucose medium (1 g L^−1^ glucose, 4.0 × 10^−3^ m l‐glutamine, without pyruvate) containing 5 × 10^−3^ m exogenous *β*‐HB, 10% fetal serum, and 1% penicillin‒streptomycin.

### Colon Cancer Tissue Microarray

A total of 180 sections of colon cancer tissue microarray (TMA: HColA180Su11) were purchased from Shanghai Xinchao Biotechnology Company, involving 93 colon cancer patients (87 pairs of cancer and adjacent tissues and 6 single cancer tissues). From November 2009 to May 2010, colon tissues were obtained by surgical resection at Taizhou Hospital, and a five‐year follow‐up visit was started from the first month after radical operation. All protocols using human specimens were approved by the Human Ethics Committee of Taizhou Hospital and Shanghai Outdo Biotech Company (YB M‐0502), and informed consent was obtained from all patients.

### Animals

The animal protocols for mouse studies were approved by the Animal Care and Use Committee of Shijitan Hospital of Capital Medical University (sjtkyll‐lx‐2020(53)). The animals received humane care according to the Guide for the Care and Use of Laboratory Animals published by the National Academy of Science and the National Institutes of Health. Aside from obesity, the animals were in generally good health.

### Diet Formulas

Please refer to **Table** **1**.

**Table 1:** Diet formulas

Composition	Standard diet formula^a)^	Ketogenic diet formula^b)^
	Mass ratio (caloric ratio)	Mass ratio (caloric ratio)
Protein	18% (0.78 kcal g^−1^)	18.2% (0.73 kcal g^−1^)
Fat	4% (0.40 kcal g^−1^)	62.8% (5.65 kcal g^−1^)
Carbohydrate	52% (2.21 kcal g^−1^)	2.6% (0.11 kcal g^−1^)
Moisture	<10%	<10%
Fiber	<5%	4.8%
Ash content	≤8.0%	5.2%
Others	Vitamins mix and minerals mix^c)^	

^a)^ Ingredients: corn, soybean meal, fish meal, flour, vegetable oil, vitamin mix, and mineral mix

^b)^ Ingredients: lard, casein, butter, corn oil, vitamin mix, mineral mix, cellulose, choline hydrogen tartrate, and dl‐methionine

^c)^ The exact compositions of the vitamin mix and mineral mix are shown in Table S1 (Supporting Information).

### Multicolor Immunohistochemistry Analysis

For mIHC staining, the instructions for the Opal Multicolor fluorescent IHC kit (PerkinElmer) were followed.^[^ ^25^ ^]^ Briefly, the TMA or slide was deparaffinized with xylol and ethanol, and antigen retrieval was performed in A6 solution (pH 6.0) by microwave. After the slide was cooled to room temperature, it was washed in TBST and blocked with blocking buffer for 10 min. Then, the tissues were incubated with primary antibody (Proteintech), followed by Opal‐HRP and Opal‐fluorogen. The above antigen retrieval and antibody incubation processes were repeated for mIHC staining. The Opal‐flurogen settings of TMA in this study were as follows: Opai‐DAPI; Opal 520‐GLUT1 (1:2000); Opal 570‐OXCT1 (1:500); Opal 620‐CK (1:1000); Opal 650‐PFKFB3 (1:3000); Opal 690‐ACAT1 (1:1000). TMA slides were scanned using a confocal microscope (Nikon, Japan), and inform software was used to analyze the images. Fluorescent signaling analysis procedure: tissue segmentation, cell recognition and segmentation, scoring cells based on intensity thresholds and marking each positive cell with a colored dot. All stained cells were observed, and the percentage of positive cells was calculated as *x*%. The view‐color settings are as follows: DAPI‐blue, GLUT1‐red; PFKFB3‐orange; CK‐magenta; OXCT1‐cyan; ACAT1‐green.

### Measurement of the OCR and Cell Energy Phenotype in Cells

The Seahorse Extracellular Flux (XF24) Analyzer (XF24, Agilent Technologies) was applied to measure the OCR (XF Cell Mito Stress Test kit, Agilent) and cell energy phenotype (XF Cell Energy Phenotype Test Kit, Agilent) of cancer cells according to the manufacturer's instructions. Briefly, the cells were plated in a Seahorse XF cell culture microplate at 3 × 10^4^ cells (SW480) or 5 × 10^4^ cells (HT29) per well, with specific treatment for 48 or 24 h before the subsequent test. The sensor cartridge was hydrated in an Agilent Seahorse XF calibrant at 37 °C in a non‐CO_2_ incubator overnight. The second day, the assay medium was prepared by supplementing Seahorse XF DMEM Base Medium according to the requirement of each teat. The assay medium was warmed to 37 °C, and the pH was adjusted to 7.4 with 0.1 N NaOH. Cell culture growth medium was removed from the cell culture microplate, and the cells were washed twice with warmed assay medium. Then, 500 µL assay medium was added, and the microplate was placed into a 37 °C incubator without CO_2_ for 1 h prior to measurement. Meanwhile, the contents of each punch of the test kit were resuspended with prepared assay medium in volumes according to the instructions.^[^ ^26^ ^]^ Loading template onto the Seahorse XF24 Analyzer and performing the appropriate procedure for analysis. The relative cell numbers of each sample were further detected by the CCK8 test, which was analyzed on a multiwell scanning spectrophotometer at 450 nm (Molecular Devices, USA).

### Western Blot

Immunoblotting was performed as previously described.^[^ ^27^ ^]^ Briefly, cells or tumor tissues were lysed in RIPA buffer, and protein extraction was carried out on ice. The protein concentration was determined by a BCA protein assay kit (Boster Biological Technology). Total protein lysate was mixed with SDS loading buffer, and proteins were separated by electrophoresis on 10% SDS‐PAGE. After that, proteins were transferred onto PVDF membranes. After blocking with 5% skim milk in TBST for 2 h, the membranes were washed and incubated sequentially with primary antibody and anti‐mouse or anti‐rabbit secondary antibody. Immunostaining with the antibody was performed using ECL western blotting reagents, and densitometric analysis of the immunoblot was carried out using Image Lab software (Bio‐Rad).

### Intracellular ATP Assay

Intracellular ATP levels were determined using an ATP detection assay kit (Beyotime Biotechnology). Briefly, the cells were seeded in six‐well plates at 1 × 10^5^ cells (SW480) or 2 × 10^5^ cells (SW620 and HT29) per well, cultured in normal medium overnight, and then left in specific treatment at the 48 or 24 h time‐point before the subsequent test. The cells were harvested in 200 µL lysis buffer on ice. Then, 100 µL ATP detection solution was added to the wells of a 96‐well plate, and empty wells around each detection space were ensured to avoid luminance disturbance. The samples were left at room temperature for 5 min, 10 µL of sample was added and mixed, and then the chemiluminescence was immediately measured by a multifunctional microplate reader (PerkinElmer). The protein concentration of each sample was detected by the BCA protein assay kit.

### Mitochondrial Membrane Potential Detection

Mitochondrial membrane potential was detected using the Mitochondrial Membrane Potential Detection assay kit (JC‐1) (Beyotime Biotechnology). The appropriate cell density (4 × 10^5^/well for SW480 cells, 8 × 10^5^/well for HT29 and SW620 cells) was applied to the six‐well plate, cultured in normal medium overnight, and then left in specific treatment at the 48 or 24 h time‐point before the subsequent test. The cells were harvested into 2 mL mixed buffer with DMEM and JC‐1 staining working solution (1:1) and incubated for 20 min in a 37 °C cell incubator for testing. After cell incubation, the supernatant was discarded, washed twice with precooled JC‐1 staining buffer, and filtered to form a single‐cell suspension. Flow cytometry (Beckman Coulter, USA) was applied to detect JC‐1 monomer (green fluorescence, FITC) and JC‐1 polymer (red fluorescence, PE) in 10 000 random cells. Relative mitochondrial membrane potential levels were calculated by the ratio of cells with red fluorescence and cells with green fluorescence (CytExpert, Beckman Coulter).

### Intracellular Acetyl‐CoA Assay

Intracellular acetyl‐CoA levels were measured by an acetyl‐CoA assay kit (Comin, Bio). The cells were seeded in six‐well plates at 1 × 105 cells (SW480) or 2 × 105 cells (HT29) per well, cultured under normal medium overnight, and then left in specific treatment at the 48 or 24 h time‐point before the subsequent test. The cells were harvested in 200 µL lysis buffer according to the instructions. Then, 184 µL acetyl‐CoA detection solution was added to the wells of a 96‐well plate and placed at room temperature for 5 min. Then, 20 µL of sample was added, and chemiluminescence was immediately measured by a multifunctional microplate reader (PerkinElmer). The values were recorded every 60 s, and the relative concentration (*y*) of intracellular acetyl‐CoA was calculated according to the maximum Δ*A* (Δ*A* = *A*_2_−*A*_1_, *y* = 1640*Δ*A* + 0.012). The protein concentration of each sample was detected by the BCA protein assay kit.

### Proliferation Assay

Cell proliferation was determined over a 5‐d period by Cell Counting Kit‐8 (CCK‐8) as described.^[^ ^28^ ^]^ The cells were seeded in 96‐well plates at 2 × 10^3^ cells (SW480) or 4 × 10^3^ cells (SW620 and HT29) per well, cultured under normal medium overnight, and then left in specific treatment, such as low‐glucose (LG), containing exogenous *β*‐HB (LG+HB), and containing p53 activator conditions (LG+COTI2), with four repeats. At each detection point, Cell Counting Kit‐8 working solution (100 µL/well) was added, and the cells were subsequently incubated for 1 h at 37 °C. Then, the plates were read on a multiwell scanning spectrophotometer at 450 nm (Molecular Devices, USA).

### Genomic DNA Extraction and Sequencing

The study sampled the tumor tissue of patient‐derived xenograft (PDx) models (50 mg) and cancer cells (1 × 10^6^) for DNA extraction. Total genomic DNA was isolated and purified according to the manufacturer's protocol using a TIANGEN TIANamp Genomic DNA Kit. The DNA sample that processed a minimum concentration of 0.1 µg µL^−1^ passed quality control with 1.8 < *A*_260/280_ < 2.0 under a NanoDrop (Thermo Fisher). KRAS and p53 primers were designed according to NCBI Primer‐BLAST for gene amplification and sequencing.

### Immunofluorescence and Confocal Microscopy

To determine the subcellular distribution of mitochondria and measure the changes in mitochondrial morphology, HT29 and SW480 cells were stained with a 200 × 10^−6^ m MitoTracker Green FM assay kit (Thermo Fisher) for 30 min at 37 °C according to the manufacturer's instructions. After washing with PBS twice, the cells were visualized under a confocal laser scanning microscope with the 488‐fluorescent channel (SIM, Nikon).

### Transmission Electron Microscopy

HT29 cells were harvested in 1 mm^3^ and fixed in 2.5% glutaraldehyde at room temperature. Next, samples were dehydrated in ethanol (with 3% uranyl acetate) and embedded in a mixture of epoxy resin and propylene oxide for 24 h. After the 70‐nm‐thick sample sections were stained with lead citrate, the mitochondrial morphology was detected under a transmission electron microscope (120 kV, TEM, FEI).

### Lentiviral Vector Construction and Infection

According to the gene information (OXCT1,3‐oxoacid CoA‐transferase1, human, Gene ID: 5019) and mRNA reference sequence (NM_000436.3) in the NCBI database, OXCT1 lentiviral shRNA constructs (with pLent‐U6‐GFP‐Puro lentiviral vector and the nonfluorescent pLent‐U6‐Puro lentiviral vector) were established (Jinkairui, Wuhan). As described,^[^ ^29^ ^]^ the lentiviral vectors were cotransfected with helper plasmids into 293T cells. The viral supernatant was collected 48 h after transfection and used to infect colon cancer cells (SW480). The fluorescence intensity was observed under a fluorescence microscope, and 6 µg mL^−1^ puromycin was added to the cells for 72 h. After the interference efficiency was verified by western blot, cell resistance was persistently maintained with a low concentration of puromycin (2 µg mL^−1^).

### ChIP and qPCR

ChIP assays were performed with a ChIP kit (Beyotime Biotechnology), and then the TIANamp Genomic DNA kit was used for DNA purification. Briefly, the cells were seeded in 10 cm culture plates at 5 × 10^5^ cells (HT29), cultured in normal medium overnight, and then left in specific treatment at the 48 or 24 h time‐point before the subsequent test. The cells were harvested after fixation with formaldehyde and then quantified into 1 × 10^6^ cells per pipe for ChIP assays. qPCR analysis was performed to detect DNA fragments immunoprecipitated with anti‐p53 antibody (1:200, Proteintech, 10442–1‐AP).

### Cell‐Derived Xenograft Assays

Colon cancer cells (HT29, p53^mt^/G^+^/K^−^) were routinely cultured to the expected number. Cells were harvested when grown to 80% confluence and resuspended in a suitable volume of fresh medium to a density of 2.5 × 10^7^ cells mL^−1^, added to a 1/5 volume of Matrigel and placed on ice for the next step. Alcohol (75%) was used to wipe the injection site of the nude mice, and 200 µL of the cell suspension was subcutaneously injected into the right side of each BALB/C‐nu nude mouse with a 1 mL syringe. Tumor formation was observed, and tumor volume was calculated by the formula (0.52 × length × width × thickness, cm^3^). The nude mice were randomly divided into four groups when the average tumor volume reached ≈0.1 cm^3^ (arm > 4). a) SD: normal diet, intraperitoneal injection of the same amount of normal saline; b) *P53* agonist (COTI2) group: normal diet, COTI2 was administered continuously for 5 d week^−1^, and intraperitoneal injection was 10 mg kg^−1^ only. COTI2 was diluted to 0.3665 mg mL^−1^ with normal saline, and the injection volume was calculated by volume. The injection volume of each nude mouse was less than 1 mL; c) KD: ketogenic feed, no calorie restriction, intraperitoneal injection of the same amount of normal saline; d) Group of ketogenic diet combined with *P53* agonist (KD+COTI2): ketogenic feed, no calorie restriction. COTI2 was injected intraperitoneally, and the amount was calculated as above. After two weeks of continuous observation, when the tumor volume reached ≈1.5 cm^3^ or the growth activity of nude mice was significantly restricted, the mice were sacrificed by sodium pentobarbital anesthesia, and the subcutaneous tumor was quickly peeled off. After rinsing with physiological saline, half of the tissue was cut and placed in liquid nitrogen, and the rest of the tissue was fixed in 4% paraformaldehyde solution and later embedded in paraffin wax to form paraffin sections.

### Immunohistochemistry

The paraffin sections were deparaffinized with xylol, ethanol, and demineralized water. Antigens were then retrieved by boiling in 1 × 10^−3^ m EDTA (pH 8.0) for 15–20 min. After the sections had been washed in demineralized water and phosphate buffered saline (PBS), the primary antibody was added and incubated for 45 min at 37 °C. After washing with PBS, the sections were incubated with EnVision Mouse or rabbit conjugate for 15 min at 37 °C. The color reaction was completed with the DAB‐positive substrate. Sections were counterstained with hematoxylin. Parallel sections were incubated with irrelevant, isotype, and concentration‐matched monoclonal antibodies as negative controls. The absence of staining due to technical failure was excluded by including appropriate positive control tissues in each staining run. Nuclear molecular expression was counted with nuclear staining according to the number of positive cells. Cytoplasmic and membranal molecular expression was analyzed according to the integrated optical density (IOD).

### Patient‐Derived Xenograft Assays

Fresh colon cancer samples obtained from patients were immediately subcutaneously inoculated into NSG mice^[^ ^30^ ^]^ to generate PDX models. After the successfully established PDX (P1) reached 1.0 cm^3^ (0.52 × length × width × thickness, cm^3^), the tissues were transplanted to the second generation (P2), and each mouse was inoculated with tissues of ≈20 mg (2 mm^3^). Eventually, the mice bearing P3 grafts were randomized into SD and KD groups for further treatment. For further KD and COTI2 investigations, mice bearing P4 grafts of PDX3 were prepared as above, and NSG mice were randomly divided into four groups when the average tumor volume reached ≈0.01 cm^3^ (arm > 4). a) SD group, normal diet, intraperitoneal injection of the same amount of normal saline; b) COTI2 group: normal diet, COTI2 was administered continuously for 5 d week^−1^, and intraperitoneal injection was 10 mg kg^−1^; c) KD group: ketogenic feed, no calorie restriction, intraperitoneal injection of the same amount of normal saline; d) KD+COTI2 group: ketogenic feed, no calorie restriction. COTI2 was injected intraperitoneally, and the amount was calculated as above. Weight, serum glucose (YAPEI, China), and serum ketone bodies (YAPEI, China) were measured, and tumor volumes were calculated as previously described. After more than three weeks of continuous observation, when the tumor volume reached ≈1.5 cm^3^ or the growth activity of nude mice was significantly restricted, the mice were sacrificed by sodium pentobarbital anesthesia, and the subcutaneous tumor was quickly peeled off. After rinsing with physiological saline, half of the tissue was cut and placed in liquid nitrogen for further analysis, and the rest of the tissue was fixed in 4% paraformaldehyde solution and later embedded in paraffin wax to form paraffin sections.

### 4D Label‐Free LC‐MS/MS Proteomics

Tumor tissues were obtained from PDX mice as described above (SD and KD groups, *n* = 3). The samples were grinded with liquid nitrogen into cell powder and dissolved in lysis buffer (8 m urea, 1% protease inhibitor cocktail), which was followed by sonication three times on ice using a high‐intensity ultrasonic processor (Scientz). The supernatant was collected after centrifugation at 12 000 *g* at 4 °C for 10 min, and the protein concentration was determined with a BCA kit. For digestion, the protein solution was reduced with 5 × 10^−3^ m dithiothreitol for 30 min at 56 °C and alkylated with 11 × 10^−3^ m iodoacetamide for 15 min at room temperature in darkness. The protein sample was then diluted by adding 100 × 10^−3^ m TEAB to a urea concentration less than 2 m. Finally, trypsin was added at a 1:50 trypsin‐to‐protein mass ratio for the first digestion overnight and a 1:100 trypsin‐to‐protein mass ratio for a second 4 h digestion. Finally, the peptides were desalted by a C18 SPE column. The tryptic peptides were dissolved in solvent A (0.1% formic acid in water) and directly loaded onto a nanoflow reversed‐phase high‐performance liquid chromatography (RP‐HPLC) analytical column (25 cm length, 75 µm internal diameter).^[^ ^31^ ^]^ Peptides were eluted at a constant flow rate of 300 nL min^−1^ using a gradient of solvent B (0.1% formic acid in acetonitrile), which ramped to 4%‐6%‐24%‐32%‐80% B at 2‐70‐84‐87‐90 min on a nanoElute high‐performance liquid chromatography (UHPLC) system (Bruker Daltonics). The peptides were subjected to capillary source followed by timsTOF Pro mass spectrometry (Bruker Daltonics) with an electrospray voltage of 1.60 kV. Precursors and fragments were analyzed at the TOF detector, with an MS/MS scan range from 100 to 1700 m/z. The timsTOF Pro was operated in parallel accumulation serial fragmentation (PASEF) mode. Precursors with charge states 0–5 were selected for fragmentation, and 10 PASEF‐MS/MS scans were acquired per cycle. The dynamic exclusion was set to 30 s.

### LC‐MS/MS Data Processing

The MS data were processed using Proteome Discoverer (v2.4.1.15) and searched in the UniProt human database (Homo_sapiens_9606_SP_20201214.fasta) while concatenating with the reverse decoy database. Trypsin (Full) was set as the cleavage enzyme, allowing up to two missing cleavages. The mass tolerance for precursor ions was adjusted to 10 ppm, and for fragment ions, it was 0.02 Da. Carbamidomethyl on Cys was set as a fixed modification, while oxidation on Met and acetylation on the protein N‐terminus were specified as variable modifications. Peptide and protein false discovery rates (FDRs) were adjusted to <1%.

### Analysis of Mitochondrial Morphology

The length of mitochondria imaged by confocal microscopy was measured using Image‐Pro Plus software. Thirty cells were randomly selected from the fields of view, and the length and width of each mitochondrion were measured. According to the percentage of mitochondrial morphology with a length/width ratio greater than 5, cells can be classified into three types: fragmented type (<10%), intermediate type (10%–30%), and tubulated type (>30%). The quantification of mitochondria photographed by TEM was measured using NIS‐Elements AR 4.5 software and measured with a length/width.

### Proteomics Analysis of Annotation and Functional Enrichment

The GO annotation proteome was derived from the UniProt‐GOA database (http://www.ebi.ac.uk/GOA/) and InterProScan software (http://www.ebi.ac.uk/interpro/), which was based on the protein sequence alignment method. Proteins were classified by Gene Ontology annotation based on three categories: biological process, cellular component, and molecular function. Functional descriptions of the identified protein domains were annotated by InterProScan software based on the InterPro domain database. Subcellular localization, such as the cytoplasm, nucleus, mitochondria, Golgi apparatus, and endoplasmic reticulum (ER), was predicted by WoLF PSORT software (https://wolfpsort.hgc.jp/). The Kyoto Encyclopedia of Genes and Genomes (KEGG) database (https://www.kegg.jp/) was further used to annotate protein pathways. In pairwise comparisons, proteins with fold changes greater than 1.5 and adjusted P values less than 0.05 were defined as differentially expressed proteins (DEPs). The functional enrichment analysis of the DEPs was performed using a two‐tailed Fisher's exact test, and the GO terms/KEGG pathway/protein domain with a corrected *p*‐value < 0.05 was considered significant. The STRING database (https://www.string‐db.org/, version 11.0) was applied for protein–protein interaction (PPI) analysis based on the accessions or sequences of DEPs. The confidence score was set as ≥ 0.7 (high confidence) to fetch all interactions, which was then visualized in the R package “networkD3.”

### Enrichment‐Based Clustering Analysis

Principal component analysis was applied to compare the difference between groups using the R function “fast.prcomp” and visualized by the R package “ggplot.” One‐way hierarchical clustering (Euclidean distance, average linkage clustering) was performed based on the functional classifications of DEPs (such as GO term, domain, and pathway). The DEPs were divided into quartiles according to their differential expression multiples (fold change, KD vs SD groups), named Q1–Q4: Q1, fold change ≤ 1/1.5; Q2, 1/1.5 < fold change ≤ 1/1.3; Q3, 1.3 < fold change ≤ 1.5); Q4, fold change > 1.5. Cluster membership was visualized by a heatmap using the “heatmap.2” function from the R package “ggplot.”

### Statistical Analysis

Each assay was conducted in at least three replicates, and values are presented as the mean ± SEM or mean ± SD. Statistical analyses were performed using GraphPad Prism software, version 8.3. Unpaired *t*‐tests were performed for comparisons between two groups. Three or more groups were analyzed using two‐way analysis of variance (ANOVA) followed by a Newman–Keuls multiple comparison test. Kaplan–Meier curves were generated by the statistical computer program SPSS statistics (IBM, version 22.0). Univariate and multivariate Cox proportional survival hazard ratios (HRs) together with 95% confidence intervals (95% Cls) were generated for prognostic analysis. Statistical significance was calculated and indicated (****P < 0.001, ***P < 0.001, **P < 0.01, and *P < 0.05).

### Reagent or Resource

Please refer to **Table** **2**.

**Table 2:** Reagent or resource

Reagents or resource	Source	Cat no.
**Antibodies**		
Anti‐*β*‐actin, dil:1/5000	ProteinTech	66009‐1‐lg
Anti‐ACAT1,dil:1/1000	ABclonal	A13273
Anti‐BDH1,dil:1/2000	ProteinTech	15417‐1‐AP
Anti‐GLUT1,dil:1/1000	ABclonal	A11170
Anti‐LDHA,dil:1/5000	ProteinTech	19987‐1‐AP
Anti‐NDUFS1,dil:1/1000	ProteinTech	12444‐1‐AP
Anti‐NDUFV1, dil:1/1000	ProteinTech	11238‐1‐AP
Anti‐NDUFV2,dil:1/1000	ProteinTech	15301‐1‐AP
Anti‐OXCT1,dil:1/1000	ProteinTech	12175‐1‐AP
Anti‐P53,dil:1/1000	ProteinTech	10442‐1‐AP
Anti‐PFKFB3,dil:1/2000	ProteinTech	13763‐1‐AP
Anti‐PGC1*α*,dil:1/1000	ProteinTech	22378‐1‐AP
Anti‐DRP1,dil:1/1000	ABclonal	A17069
Anti‐phospho‐DRP1(S616),dil:1/1000	ABclonal	AP0849
Anti‐phospho‐DRP1(S637),dil:1/1000	ABclonal	AP0812
Anti‐Ki67,dil:1/50	Servicebio	GB11030
Anti‐PCNA,dil:1/50	Servicebio	GB11010
Goat anti‐mouse IgG‐HRP,dil:1/10000	Jackson	115‐005‐003
Goat anti‐rabbit IgG‐HRP,dil:1/10000	Jackson	111‐005‐003
**Chemicals, peptides, and recombinant proteins**		
Dulbecco's modified Eagle's medium (DMEM)	HyClone	SH30243.01
Low‐glucose Dulbecco's modified Eagle's medium (LG‐DMEM)	GENOM	GNM31672
Nonglucose Dulbecco's modified Eagle's medium (NG‐DMEM)	Gibco	11966025
Fetal bovine serum (FBS)	HyClone	SH30084.03
Puromycin	Gibco	A1113803
Trypsin	Boster Biol Tech	AR1007
1% penicillin/streptomycin	Boster Biol Tech	AR1203
DMSO	Sigma	D2650
d‐glucose	Sigma	D8270
ECL	Pierce	23891
l‐glutamine	Sigma	G3126
Sodium pyruvate solution	Sigma	S8636
TRIzol	Invitrogen	T9424
RIPA	Boster Biol Tech	AR0102
PMSF	Boster Biol Tech	AR1192
SDS‐PAGE protein loading buffer	Boster Biol Tech	AR1112
XF DMEM base medium, pH 7.4, 500 mL	Agilent technologies	103575‐100
Antifluorescent quench sealant	Southernbiotech	32352
Phosphate buffer	Boster Biol Tech	AR1201
Skimmed milk powder	Becton,Dickinson	12424
DAPI dye	Beyotime Biotechnology	C1005
Paraffin	Sinopharm	3112
Anhydrous ethanol	Sinopharm	3214
2.5% glutaraldehyde	Solarbio	CP1126
Xylene	Sinopharm	3452
**Critical commercial assays**		
Opal‐7 multicolor fluorescent IHC kit	PerkinElmer	NEV20000
BCA Protein assay kit	Boster Biol Tech	AR0197
CCK‐8 assay kit	Beyotime Biotechnology	C0037
qPCR assay kit	Vazyme Biotech	R122‐01
XF Cell Mito Stress Test kit	Agilent technologies	103015‐100
XF Cell Energy Phenotype Test Kit	Agilent technologies	103325‐100
ATP Detection assay kit	Biotime Biotechnology	S0026
XF Cell Culture Microplates	Agilent technologies	Lot: 33818
XF Extracellular Flux Assay Kit	Agilent technologies	Lot: B29018
Mitochondrial membrane potential assay kit (JC‐1)	Beyotime Biotechnology	C2006
Acetyl‐CoA Assay Kit	Comin Biotech	ACA‐1‐Y
TIANamp Genomic DNA Kit	TIANGEN BIOTECH, BEIJING	DP304
ChIP Assay Kit	Beyotime Biotechnology	P2078
MitoTracker Green FM	Thermo Fisher	M7514
**Others**		
Mouse normal diet	BEIJING HFK BIOSCIENCE	1022
Mouse ketogenic diet	Jielikang Biotech	1025
PVDF membrane	Mmillipore	IPVH00010
**Experimental models: organisms/strains**		
BALB/C‐nu mice	Vital River	N/A
NSG (NPI. NOD‐Prkdc^scid^‐IL2rg^emlIDMO^) mice	IDMO Co.Ltd, Beijing	N/A
**Deposited Data**		
Publicly available transcriptomic datasets from colon cancer patients	GEO database	GSE17536 GSE17537 GSE39582
Publicly available transcriptomic datasets from colon cancer tissue	TCGA database	https://cancergenome.nih.gov/
Experimental models: cell lines		
Human:HT29	Procell Life Sci Tech	N/A
Human:SW480	Procell Life Sci Tech	N/A
Human:SW620	Procell Life Sci Tech	N/A
Software and algorithms		
Graphpad Prism 8	GraphPad	https://www.graphpad.com/scientific‐software/prism/
SPSS statistics 19.0	IBM Corporation	https://www.ibm.com/analytics/spss‐statistics‐software/
CytExpert	Beckman Coulter	https://www.beckmancoulter.cn/
Image Lab	Bio‐Rad	https://www.bio‐rad.com/

Oligonucleotides	Source	Cat no.
shRNA1 targeting sequence: OXCT1 GCCAATGCAGCAGATCGCAAATCTCGAGATTTGCGATCTGCTGCATTGGTTTTT	Gene Create	N/A
shRNA2 targeting sequence: OXCT1 GCCTGGTACAAGGGATGTGTTTCTCGAGAAACACATCCCTTGTACCAGGTTTTT	Gene Create	N/A
shRNA3 targeting sequence: OXCT1 GACCGAGCAGGAAACGTGATTTCTCGAGAAATCACGTTTCCTGCTCGGTTTTTT	Gene Create	N/A
p53 primer‐ex2 (439 bp) 2F: TCAGACACTGGCATGGTGTTG; 2R: ATGGAATTTTCGCTTCCCAC	Taihe Biotech	N/A
p53 primer‐ex3‐4 (536 bp) 3F: ACTTTCTGCTCTTGTCTTTCAG; 4R: AGCCAAAGGGTGAAGAGGAATC	Taihe Biotech	N/A
p53 primer‐ex5‐6 (590 bp) 5F: AGGAGGTGCTTACGCATGTTTG; 6R: AGGGAGGTCAAATAAGCAGCAG	Taihe Biotech	N/A
p53 primer‐ex7 (304 bp) 7F: AGGAGGTGCTTACGCATGTTTG; 7R: AGGGAGGTCAAATAAGCAGCAG	Taihe Biotech	N/A
p53 primer‐ex8‐9 (491 bp) 8F: AGGAGGTGCTTACGCATGTTTG; 9R: AGGGAGGTCAAATAAGCAGCAG	Taihe Biotech	N/A
p53 primer‐ex10 (315 bp) 10F: GCATGTTGCTTTTGTACCGTC; 10R: AGCTGCCTTTGACCATGAAG	Taihe Biotech	N/A
p53 primer‐ex11 (274 bp) 11F: AGCCTTAGGCCCTTCAAAGC; 11R: TGCAAGCAAGGGTTCAAAGAC	Taihe Biotech	N/A
OXCT1‐promoter primer‐site1 F: gTGTTTTGACCATACAGTAATTAGgg; R: gGGTCTGCTTTTCCACTTTTGccct	Tsingke Biotech	N/A
OXCT1‐promoter primer‐site2 F: CAGAGTTAAAAGAATACCATGAAagggc; R: ggaaAGACCAAGTGGAAGCTCAATAGCA	Tsingke Biotech	N/A
OXCT1‐promoter primer‐site3 F: AAAAGCAACAAGCTTGGCGGGAGATcc; R: AAGTTCCTCTCAGCGCCTCAggtg	Tsingke Biotech	N/A
BDH1‐promoter primer F: tctgagccagatcaatttaggaag; R: acctttccagactgagccaatg	Tsingke Biotech	N/A
ACAT1‐promoter primer F: gcactgaggggagttgcttg; R: aagaacgggctaatgaaggcac	Tsingke Biotech	N/A

## Conflict of Interest

The authors declare no conflict of interest.

## Author Contributions

M.T., H.X., H.H., and H.K. contributed equally to this work. M.T. and H.X. designed and performed the experiments, analyzed the data, and wrote the manuscript. X.‐W.Z., Q.‐Q.L., Y.‐B.C., and J.‐L.K. assisted with the animal experiments. M.T., B.Z., Y.‐Z.G., and W.‐F.Z. analyzed transcriptomic and sequencing data. M.‐M.S. and C.‐X.W. assisted with the molecular experiments. H.K., H.‐Y.H., Y.W., C.‐B. M., X.‐B.W., M.‐H.C., X.Z., G.M., and H.‐P.S. provided constructive suggestions for the project design and edited the manuscript. F.‐X.Z. and Q.S. designed the project, prepared the figures, wrote the manuscript, shaped the concept, and supervised the revision.

## Materials Availability

This study did not generate new unique reagents.

## Data and Code Availability

The comparison of transcriptome data between colon cancer and noncancer samples was formatted from the GEPIA network (http://gepia.cancer‐pku.cn/), which is based on TCGA database (The Cancer Genome Atlas, https://www.cancer.gov), including mRNA expression information of 275 colon cancer tissues and 41 colon nontumor tissues.

The transcriptome database containing prognostic information included in this study was from GEO (Gene Expression Omnibus, https://www.ncbi.nlm.nih.gov/geo/), GPL570 platform, including three data sets GSE17536 (172 cases), GSE17537 (55 cases), and GSE39582 (555 cases), and normalized by R language to obtain gene mRNA expression information of 782 colon cancer tissues. The clinical data included were age, sex, TNM stage, and overall survival.

## Additional Information

Further information and requests for resources and reagents should be addressed to the Lead Contact.

## Supporting information

Supporting Information

## Data Availability

The data that support the findings of this study are available from the corresponding author upon reasonable request.
