# Supplementary material for: Metabolism‐Based Molecular Subtyping Endows Effective Ketogenic Therapy in p53‐Mutant Colon Cancer
Source: Adv Sci (Weinh). 2022 Aug 28;9(29):2201992. doi: 10.1002/advs.202201992 (PMC9561794; doi:10.1002/advs.202201992)
Supplement: Supplementary file 1 — Supporting Information [file ADVS-9-2201992-s001.pdf]

Table S1. The exact composition of the vitamin and mineral mix.

Composition	Value
**Vitamins**	
Vitamin A	13800 U
Vitamin B1	11.4 mg
Vitamin B2	11.5 mg
Vitamin B6	11 mg
Vitamin B12	19 mcg
Ascorbic Acid	0 mg
Vitamin D3	1900 U
Vitamin E	220 U
Vitamin K3	2 mg
Niacinic acid	57.1 mg
Pantothenic acid	28 mg
Biotin	0.38 mg
Choline	1790 mg
Folic acid	3.8 mg
**Minerals**	
Calcium	9.9 gm
Phosphorus	6.6 gm
Chloride	3.0 gm
Copper	11.5 mg
Chromium	3.8 mg
Fluoride	0.0 mg
Iodine	0.4 mg
Iron	68.1 mg
Magnesium	1.0 gm
Manganese	111 mg
Potassium	6.9 gm
Se	0.33 mg
Sodium	1954 mg
Sulphur	641 mg
Zinc	63.7 mg

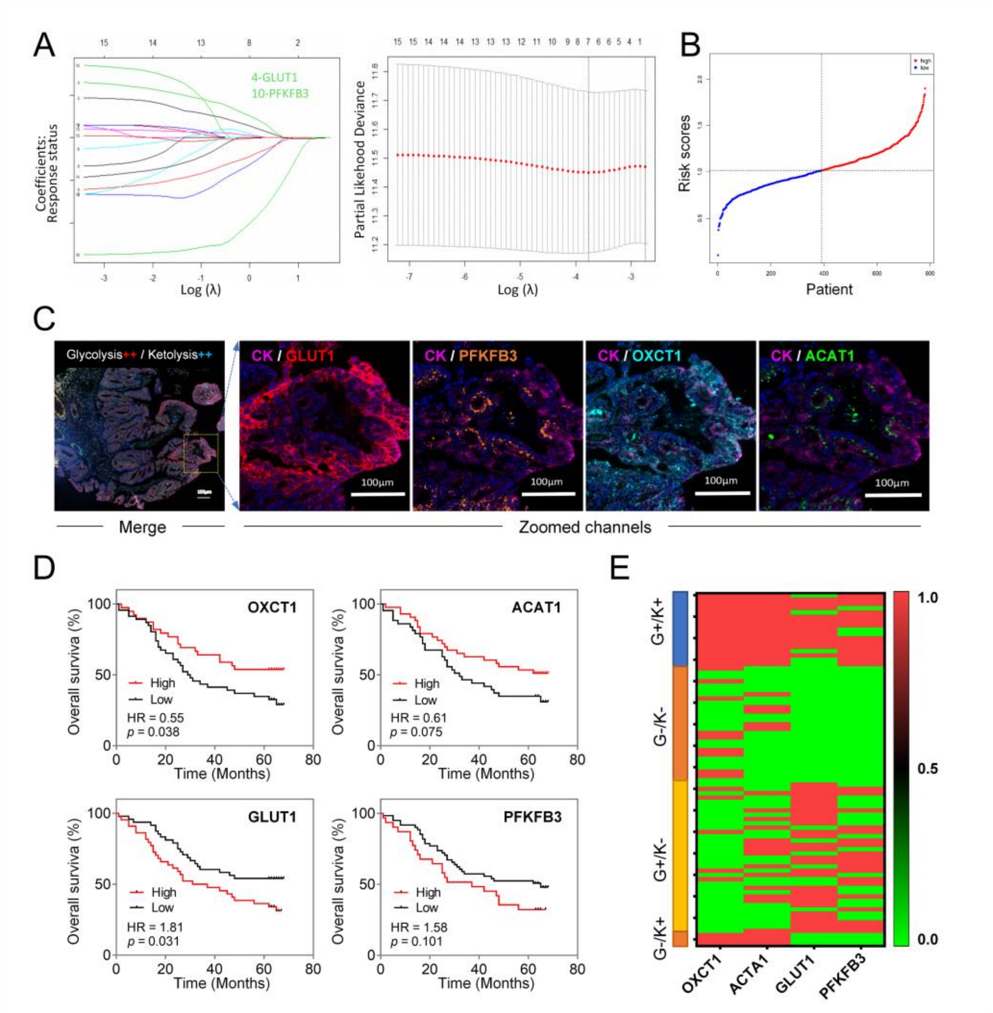

Figure S1. Metabolic molecule screening and survival analysis. Related to Figure 1.

(A) Multivariate analysis of 16 genes and cross-validation based on LASSO regression (Genes 1-16 refer to OXCT1, ACAT1, BDH1, GLUT1, HK2, PKM, MCT4, PGC1a, PFKM, PFKFB3, PDK1, LDHA, BNIP3, PDH, SIRT1, and SIRT3, respectively).

(B) Risk scores of the four-gene signature in the entire discovery set (GSE39582, GSE17536, and GSE17537).

(C) Representative images of multiplexed immunofluorescence staining (Opal-7) in colon cancer tissue microarrays. Blue indicates DAPI (nuclear staining), magenta indicates CK (epithelium), red-GLUT1, orange- PFBFK3, cyan-OXCT1, and green-ACAT1.

(D) Kaplan–Meier curve and Cox regression analysis of overall survival (OS) for metabolic molecules of colon cancer patients (OXCT1 HR=0.55, 95% CI: 0.32-0.96, P=0.038; ACAT1 HR=0.61, 95% CI: 0.35-1.06, P=0.075; GLUT1 HR=1.81, 95% CI: 1.04-3.13, P=0.031; PFKFB3 HR=1.58, 95% CI: 0.87-2.86, P=0.101).

(E) Detailed phenotype scatter with genes differentially expressed in the colon cancer microarray.

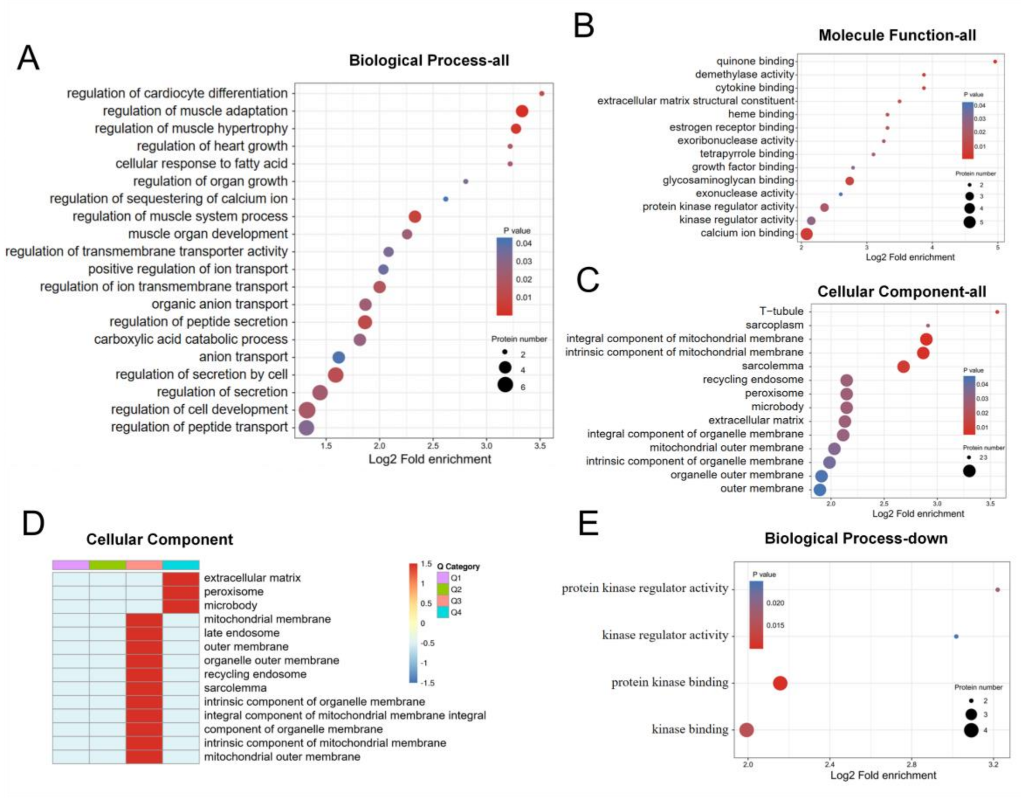

Figure S2. Differentially expressed proteins (DEPs) between the KD and SD groups. Related to Figure 3.

(A-C) Functional enrichment of differentially expressed proteins according to GO “biological process” (A), “molecular function” (B), and “cellular component” (C) between the KD and SD groups (KD/SD).

(D) Functional enrichment and cluster analysis of differential proteins according to GO “cellular component” between the KD and SD groups (KD/SD). Q1, fold change ≤ 1/1.5; Q2, 1/1.5 < fold change ≤ 1/1.3; Q3, 1.3 < fold change ≤ 1.5; Q4, fold change > 1.5.

(E) Functional enrichment of downregulated proteins according to GO “biological process” between the KD and SD groups (KD/SD).

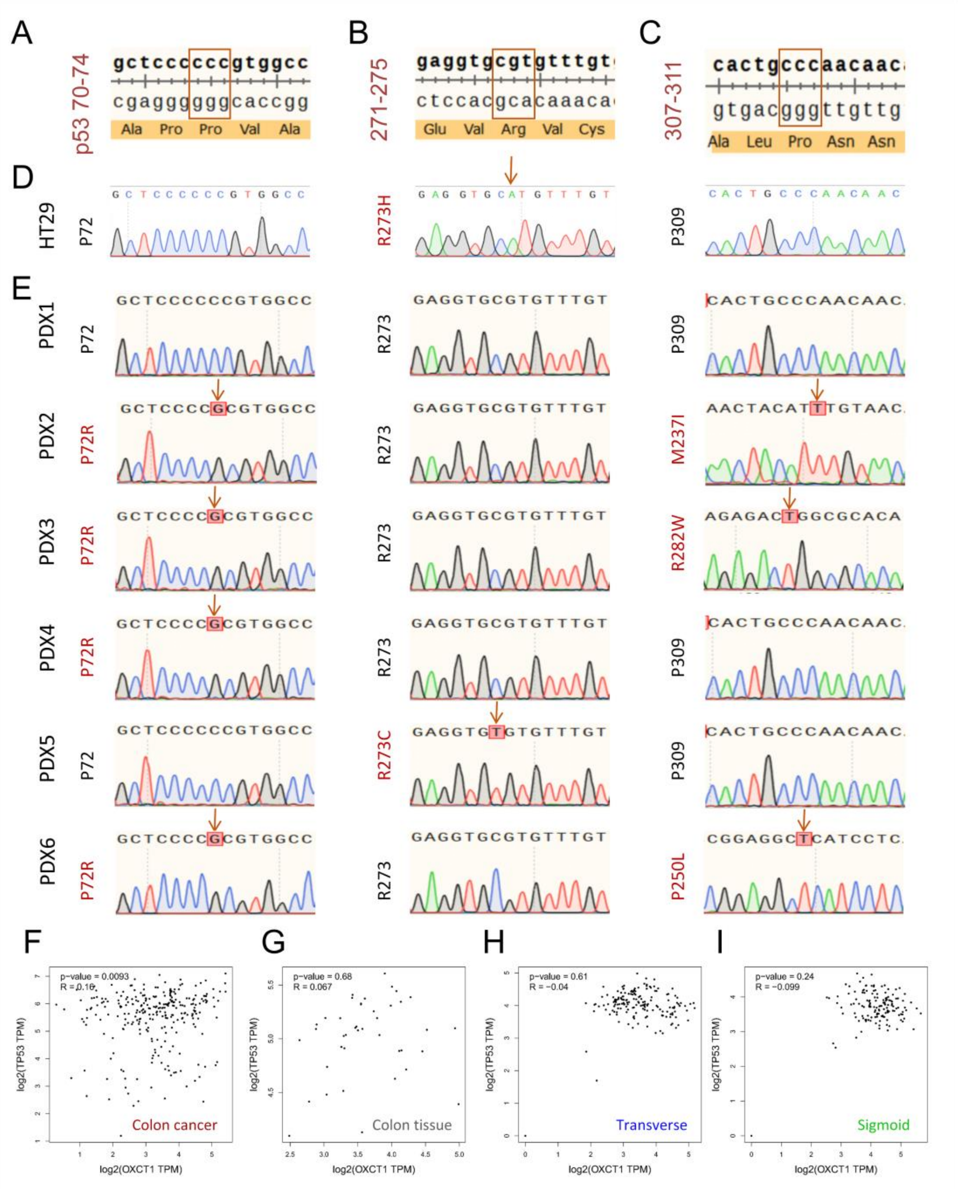

Figure S3. Mutation detection of different colon cancer cells and tissues. Related to Figure 5.

(A-C) SnapGene was used to perform the comparison of DNA sequences based on original wild-type p53. Schematic diagram of amino acids 70-74 (A), amino acids 271-275 (B), and amino acids 307-311 (C).

(D) P53 DNA sequence of HT29 cells exhibiting p53 mutations of R273H, whereas P72 and P309 were wild type.

(E) P53 DNA sequence of PDX1 tissue without mutation, while the PDX (2-6) tissues exhibited p53 mutations of P72R, M237I, R282W, R273C, and P250L.

(F-I) Correlation between the transcriptional expression of p53 and OXCT1 in colon cancer tissues (F), normal colon tissues (G), transverse colon tissues (H), and sigmoid colon tissues (I) through the GEPIA network.

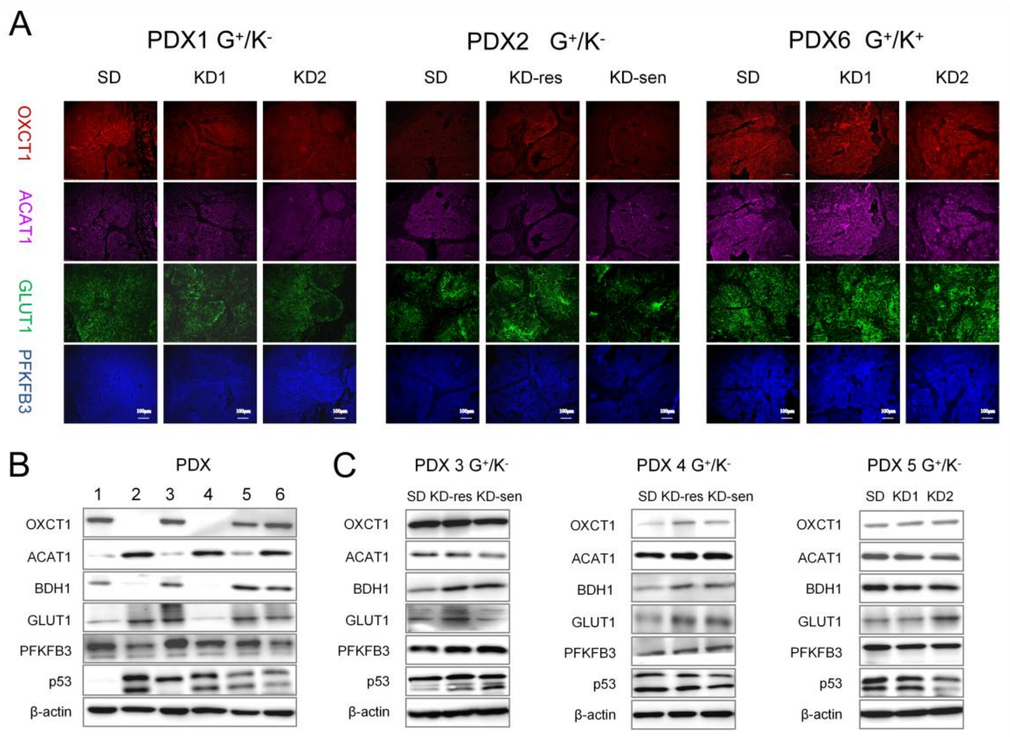

Figure S4 Molecular verifications of PDX tumor tissues. Related to Figures 2 and 5.

(A) Multiplexed immunofluorescence staining (Opal-4) of colon cancer tissues from corresponding PDx sections. Red indicates OXCT1, magenta indicates ACAT1, green-GLUT1, and blue-PFKFB3.

(B) Western blot of glycolytic and ketolytic molecules in PDX tumor tissues of the P3 generation. β-actin was used as a control.

(C) Western blot analysis of glycolytic and ketolytic molecules in corresponding PDX tumor tissues group-treated under SD and KD. β-actin was used as an internal control. PDX, patient-derived xenograft model. G^+/K^-, glycolysis$^+$/ketolysis$^-$, G^+/K^+, glycolysis$^+$/ketolysis$^+$. SD, standard diet. KD, ketogenic diet. KD-res, KD-resistant. KD-sen, KD-sensitive.

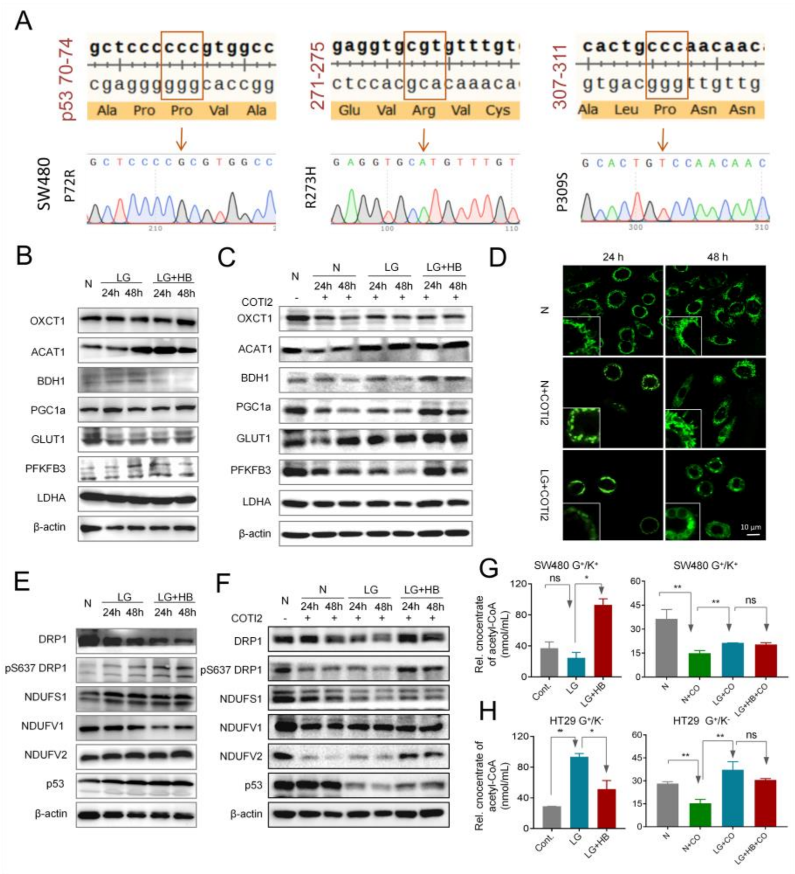

Figure S5. COTI2 reversed mitochondrial OXPHOS adaptation in p53^{mt} cells. Related to Figure 6.

(A) Schematic diagram of p53 DNA sequences at amino acids 70-74, amino acids 271-275, and amino acids 307-311 according to SnapGene. SW480 cells exhibiting p53 mutations of P72R, R273H, and P309S.

(B) Western blot analysis of glycolytic and ketolytic enzymes in SW480 cells cultured under N, LG and LG+HB conditions for 24 h and 48 h. β-actin was used as an internal control.

(C) Western blot analysis of glycolytic and ketolytic enzymes in SW480 cells cultured under N, N+COTI2, LG+COTI2 and LG+HB+COTI2 conditions for 24 h and 48 h. β-actin was used as an internal control.

(D) Mitochondrial morphology of SW480 cells cultured under N, N+COTI2, and LG+COTI2 conditions for 24 h and 48 h stained with MitoTracker (green).

(E) Western blot analysis of mitochondrial dynamin-related proteins and subunits of respiratory chain complex I in SW480 cells cultured under N, LG and LG+HB conditions for 24 h and 48 h. β-actin was used as an internal control.

(F) Western blot analysis of mitochondrial dynamin-related proteins and subunits of respiratory chain complex I in SW480 cells cultured under N, N+COTI2, LG+COTI2 and LG+HB+COTI2 conditions for 48 hours. β-actin was used as the internal control.

(G-H) The relative concentration of cellular acetyl-CoA levels in SW480 and HT29 cells cultured under N, LG, LG+HB, N+COTI2, LG+COTI2 and LG+HB+COTI2 conditions for 24 h. Values were normalized to the cellular protein levels. Data are presented as the mean ± SD. Error bars denote the SD of triplicates. $^{*}P < 0.05$ compared among each group. $^{*}P<0.05$,

**P<0.01.

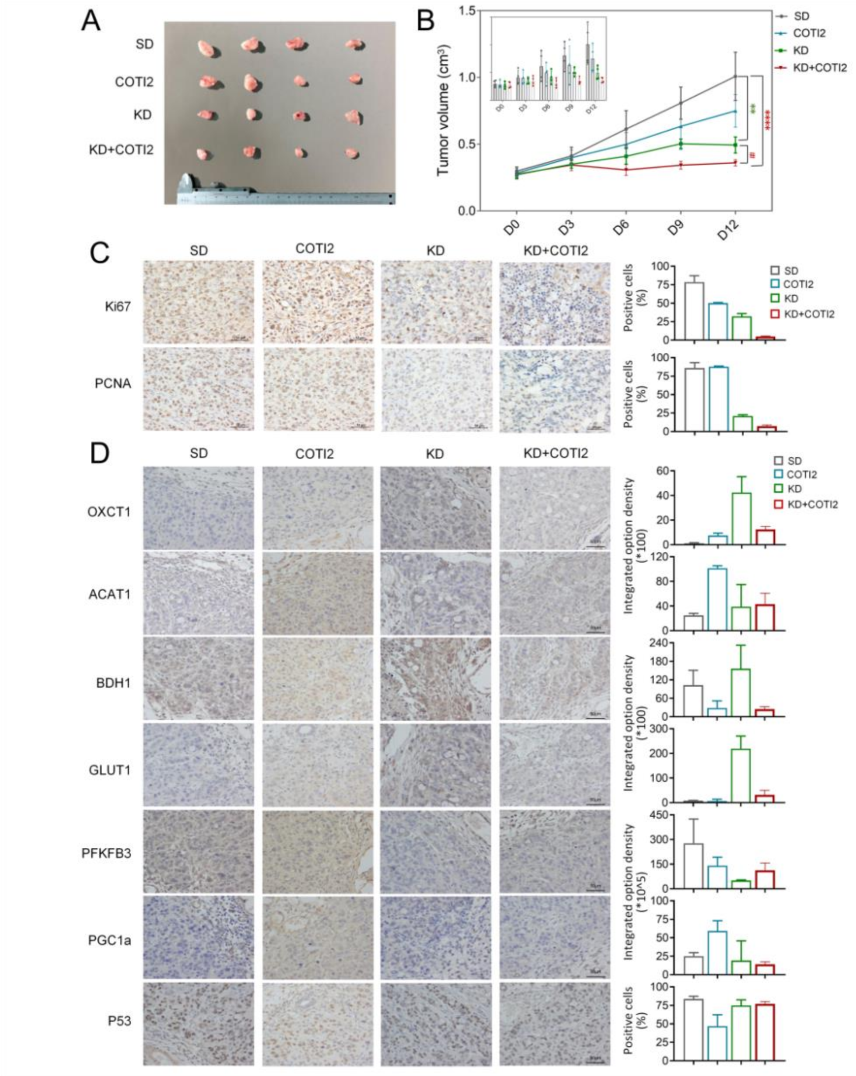

Figure S6. Reversing metabolic reprogramming promotes sensitivity to ketogenic treatment in the HT29 CDX model. Related to Figure 7.

(A-B) Tumor images (A) and volumes (B) of HT29 xenografts after 12 days of treatment with the standard diet (SD), ketogenic diet (KD), 10 mg/kg COTI2 (COTI2), or KD combined with 10 mg/kg COTI2 (KD+COTI2). Data are the mean ± SEM, and two-way ANOVA was performed among each group (n=4 mice/arm). *P < 0.05 compared between the COTI2, KD or KD+COTI2 group and the SD group, the green asterisk indicates the KD and SD groups, the red asterisk indicates the KD+COTI2 and SD groups, $^{\#}$P < 0.05 compared between the KD+COTI2 and KD groups. $^{\#}$, *P<0.05, $^{\#\#}$, **P<0.01, $^{\#\#\#}$, ***P<0.001, ****P<0.0001.

(C) Detection of proliferation markers (Ki67, PCNA) in tumor tissues from each treatment group.

(D) Histology and quantification of OXCT1, ACAT1, BDH1, GLUT1, PFKFB3, PGC1a and P53 in tumors from each group. Quantification is depicted as the score per high-powered field, and 3 images were taken for each of the four mice. P values from two-sided t tests comparing the blinded scoring in the KD group and KD+COTI2 group were 0.002, 0.994, 0.0366, 0.000,

0.784, 0.974 and 0.784 for OXCT1, ACAT1, BDH1, GLUT1, PFKFB3, PGC1a and P53, respectively.
